# Systematic comparison of variant calling pipelines using gold standard personal exome variants

**DOI:** 10.1038/srep17875

**Published:** 2015-12-07

**Authors:** Sohyun Hwang, Eiru Kim, Insuk Lee, Edward M. Marcotte

**Affiliations:** 1 Center for Systems and Synthetic Biology, Institute for Cellular and Molecular Biology, University of Texas at Austin, TX 78712, USA; 2 Department of Biotechnology, Yonsei University, Seoul, 120-749, Korea

## Abstract

The success of clinical genomics using next generation sequencing (NGS) requires the accurate and consistent identification of personal genome variants. Assorted variant calling methods have been developed, which show low concordance between their calls. Hence, a systematic comparison of the variant callers could give important guidance to NGS-based clinical genomics. Recently, a set of high-confident variant calls for one individual (NA12878) has been published by the Genome in a Bottle (GIAB) consortium, enabling performance benchmarking of different variant calling pipelines. Based on the gold standard reference variant calls from GIAB, we compared the performance of thirteen variant calling pipelines, testing combinations of three read aligners—BWA-MEM, Bowtie2, and Novoalign—and four variant callers—Genome Analysis Tool Kit HaplotypeCaller (GATK-HC), Samtools mpileup, Freebayes and Ion Proton Variant Caller (TVC), for twelve data sets for the NA12878 genome sequenced by different platforms including Illumina2000, Illumina2500, and Ion Proton, with various exome capture systems and exome coverage. We observed different biases toward specific types of SNP genotyping errors by the different variant callers. The results of our study provide useful guidelines for reliable variant identification from deep sequencing of personal genomes.

Recent advances in next generation sequencing (NGS) technology have enabled many researchers access to affordable whole exome or genome sequencing (WES or WGS), leading to the phenomenal achievements in genome sequencing projects such as the personal genome project^1^,^2^ and 1000 genome project^3^. Genome sequencing projects for healthy and disease cohorts have identified numerous functional or disease-associated genomic variants, which can give clues about therapeutic targets or genomic markers for novel clinical applications. Already many Mendelian disease studies have employed NGS to identify causal genes based on patient-specific variants^4^,^5^,^6^,^7^,^8^,^9^,^10^. Because Mendelian disease associated variants with high functional impact rarely occur among the healthy population, interpretation of patient-specific variants is relatively simple. This interpretation simplicity by virtue of rarity of the variants, however, entails risk of false discoveries due to the errors in sequencing and false detection by variant calling methods. The accurate identification of genomic variants is therefore critical factor for the success of clinical genomics based on NGS technology.

Genomic variants, such as single nucleotide polymorphisms (SNPs) and DNA insertions and deletions (also known as indels), are identified by various analysis pipelines with combinations between short read aligners and variant callers. Among many read aligners, BWA-MEM^11^, Bowtie2^12^, and Novoalign (http://novocraft.com/) are popular, and among many variant callers, the Genome Analysis Tool Kit HaplotypeCaller (GATK-HC)^13^, Samtools mpileup^14^, Freebayes^15^, and Torrent Variant Caller (TVC) are widely used in genomic variant analyses. The first three callers can be applied to both Illumina and Ion Proton sequencing data, but TVC was developed as an Ion Proton specific caller. Several studies have been conducted to compare different variant calling pipelines, and they reported substantial disagreement among variant calls made by different pipelines, suggesting a need for more cautious interpretation of called variants in genomic medicine^16^,^17^,^18^. The low concordance of variant-calling pipelines also prompted the clinical genomics community to seek for standardization of performance benchmarking of the pipelines^19^.

A systematic comparison of variant calling performance requires a gold standard set of reference variant calls. Recently, the Genome in a Bottle (GIAB) consortium published a set of highly confident variant calls for one individual in the 1000 Genome project (sample NA12878)^19^. They integrated fourteen variant data sets from five NGS technologies, seven read mappers and three variant calling methods, and manually arbitrated between discordant data sets. They also provided more highly confident calls and regions by integration of the version v2.19 GIAB calls, genomic information of pedigrees of NA12878, and Illumina Platinum project variant calls. This highly accurate and presumably mostly unbiased set of SNP and indel genotype calls for NA12878 is the only gold standard variant genotype data set publicly available for systematic comparisons of variant callers.

Recently two published studies used the GIAB variant data for comparisons of variant calling pipelines^20^,^21^. They compared multiple variant calling pipelines based on positive predictive value; (PPV; also known as precision) and sensitivity (also known as recall) for a single sequence data set. Importantly, results from these analyses indicated significant variation across the pipelines, suggesting the need for a more detailed analysis. In particular, several aspects were important to address. First, previous studies analyzed only a single data set. Thus, data-specific effects could not be excluded. To draw a conclusion that can be generalized to many personal genomes, there is a need to evaluate variant calling pipelines based on multiple data sets by various sequencing platforms, exome capture systems, and exome coverage. Second, these studies measured PPV and sensitivity, separately, to benchmark performance. Thus, a difference in false positive rate between high score variants and low score variants was not reflected in a single benchmarking score, such as, the area under a precision-recall curve (APR), which reflects the intrinsic trade-off between precision (i.e., PPV) and recall (i.e., sensitivity), providing a more informative performance score.

In this study, we compared thirteen pipelines that consist of a combination between three popular read aligners, BWA-MEM^11^, Bowtie2^12^, and Novoalign (http://novocraft.com/), and four popular variant callers, GATK-HC^13^, Samtools^14^, Freebayes^15^, and TVC, on multiple genomic sequence data sets by three NGS platforms, Illumina HiSeq2000, HiSeq2500, and Ion Proton. The NA12878 benchmark variant set was used to compare performance, concordance, and types of SNP calling errors. Because the twelve data sets selected reflect diverse sequencing platforms, exome capture systems and exome coverage, these benchmarking results should in principle be more reliably extended to personal genomics data for clinical applications. We observed that Samtools with BWA-MEM performed best for SNPs on Illumina data, while GATK-HC combined with any read aligner outperformed all other pipelines for indels. For Ion Proton data, Samtools outperformed all others including TVC, the variant caller specific for Ion Proton. From analyses of concordance among the variant calling pipelines based on high confidence variants, we observed high concordance for Illumina platforms, but low concordance for Ion Proton, and found that data set also affects concordance levels. In addition, we observed different biases toward types of SNP calling errors across the callers: Freebayes is biased toward ignoring the reference allele (IR), whereas GATK-HC and Samtools are biased toward adding the reference allele (AR).

## Results

### Sequence data sets and variant calling pipelines for this study

**Figure 1:**
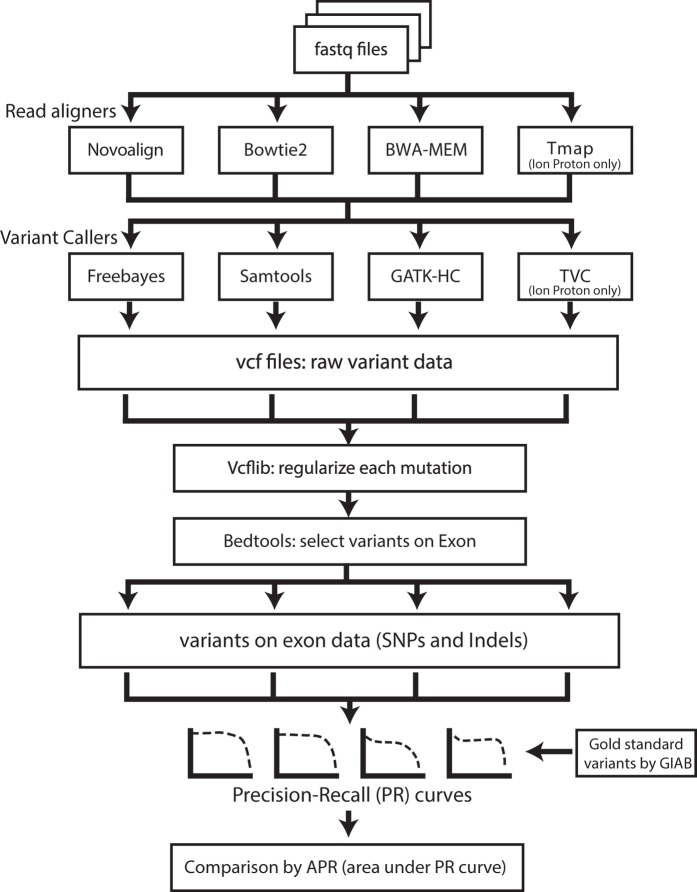
A flow diagram summarizing the performance comparison of thirteen variant calling pipelines.

Using the analysis pipeline summarized in Fig. 1, short reads of each data set were aligned by three popular aligners: BWA-MEM^11^, Bowtie2^12^, and Novoalign (http://novocraft.com/). We downloaded NA12878 sequence data sets generated by Illumina HiSeq2000, HiSeq2500, Ion Proton from SRA^22^ and GIAB FTP^19^. To reduce bias by sample effect on performance measures, we used multiple data sets of NA12878 for each Illumina platform (Table 1): seven data sets for HiSeq2000 and four data sets for HiSeq2500, with variations in the choice of whole genome sequencing (WGS) or whole exome sequencing (WES), exome capture systems, and exome coverages. However, we could analyze only one data set for Ion Proton, as only a single Ion Proton sequence data set for NA12878 was available by the time of this study. Data sets for Illumina were mapped to the Genome Reference Consortium human genome build 37 (GRCh37), using bwa-mem-0.7.10, bowtie2-2.2.25, and novoalign-v3.02.12. The downloaded data set for Ion Proton, which was pre-mapped to the GRCh37 by Tmap. Then we analyzed each aligned data set using four popular variant callers: GATK-HC^13^, Samtools^14^, Freebayes^15^ for all data sets and TVC for the Ion Proton data set only. According to the recommended procedures of each variant caller, we also removed duplicates or realigned indel regions. After running the four variant callers for each of twelve data sets, we regularized the different variant calls to the same format.

### Performance comparison of variant callers in exon regions

Performance comparison among different variant callers requires a gold standard set of variant calls. We used a set of high-confident SNP and indel calls for NA12878 by GIAB consortium^19^. We defined true positives (variants called by a variant caller as the same genotype as the gold standard data), true negatives (reference alleles in high confident regions other than gold standard variants), false positives (variants called by a variant caller but not in gold standard variant set), false negatives (gold standard variants that were not called by a variant caller), and SNP genotyping errors (variants called by a variant caller as different genotypes from the gold standard data). We employed precision-recall curves, which are known to be more informative than receiver operating characteristic (ROC) curves when the negative set is disproportionate to the positive set^23^,^24^, as a measure of variant calling performance. We sorted each called variant according to its Phred-scaled quality score and measured the performance of each variant caller. Precision-recall curves were generated for each variant caller for SNPs and indels in each of the twelve sequence data sets based on gold standard variant calls (Supplementary Figure 1). A precision-recall curve was also summarized as an area under the precision-recall curve (APR) score (Supplementary Tables 1 and 2).

**Figure 2:**
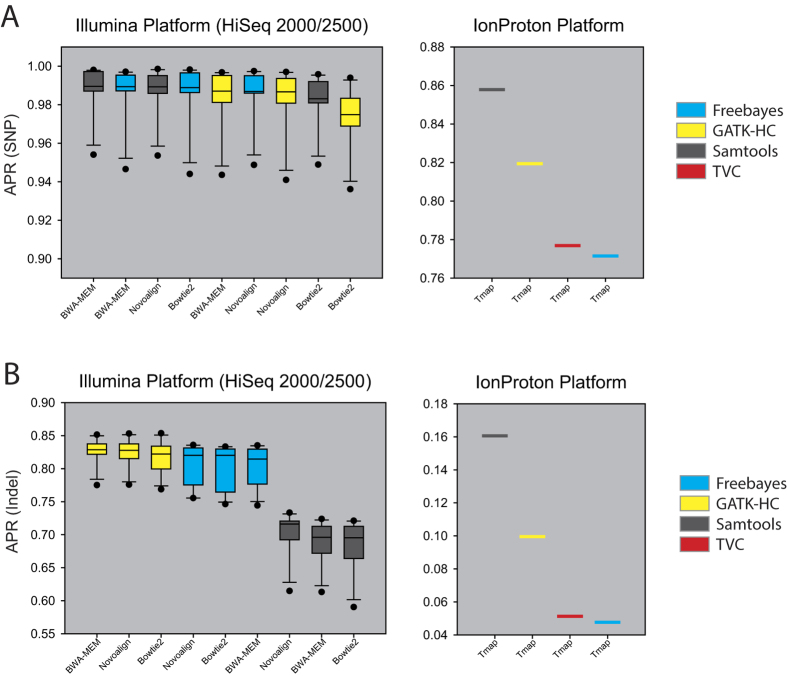
Summary of variant calling performances by thirteen pipelines. Performance of variant calling pipelines measured by APR for (**A**) SNP and (**B**) indel for multiple Illumina data sets and represented as a box plot. For Ion Proton, an APR for single data set for each of callers are indicated. Ion Proton data were pre-aligned by Tmap.

To compare the overall performance among the thirteen pipelines, we compared the distributions of APR scores of multiple data sets for each pipeline on SNPs and indels (Fig. 2). For SNP variant calls, BWA-MEM-Samtools pipeline showed the best performance and Freebayes showed good performance across all aligners for both Illumina platforms. For Ion Proton data, Samtools outperformed all other callers, including TVC, which is the Ion Proton’s own variant calling method (Fig. 2A). Interestingly, the best variant caller of each data set varies (see APR scores in Supplementary Tables 1 and 2). This observation of variation in best performed pipelines across data sets clearly demonstrates a data-specific effect of benchmarking results. Therefore, benchmarking performance of each variant calling pipeline needs to be based on multiple data sets to avoid misleading conclusions. The tested variant pipelines showed larger performance difference in calling indels. For indel calls, GATK-HC with any aligner outperformed Freebayes and Samtools on both Illumina platforms, while Samtools performed best on Ion Proton data (Fig. 2B). We did not compare performances between platforms, because the purpose of this study is to compare performance of variant callers for each sequencing platform. In addition, the Ion Proton data set has much lower exome coverage (<10×) than those of Illumina data sets (43.6×–298.5×) (see Table 1). Although TVC is the official variant caller for Ion Proton data, it performed no better than other callers on both SNPs and indels. However, we could not predicate the TVC performance based on only this result, because we tested only one Ion Proton data set for NA12878 with low exome coverage. We could perform further benchmarking studies using additional data sets for NA12878 sequenced by Ion Proton in the future to further evaluate the TVC performance.

We also investigated whether the read aligner or variant caller have more influence on the performance of variant identification. We calculated average standard deviations of APR of data sets with different aligners but the same variant caller, and those with different variant callers but the same aligner. The average standard deviation among data sets with different aligners and those with different variant callers are 3.46e-3 and 4.02e-3 for SNPs, and 0.72e-2 and 7.2e-2 for indels, respectively. These results indicate that variant caller has more influence than read aligner on both SNP and indel identifications, with a much larger effect on indel identification.

### Concordance of the four variant callers

**Figure 3:**
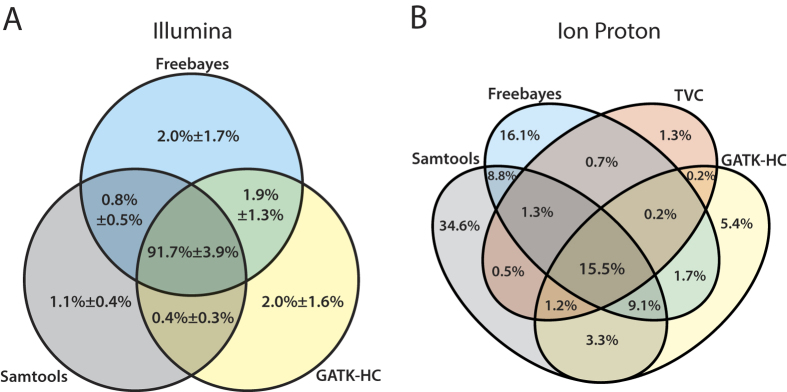
Venn diagrams summarizing called variants by different callers. The mean percentage with standard deviation of confidence variant calls with equal to or higher than the quality score threshold of 20 are represented for (**A**) Illumina data sets and (**B**) Ion Proton data set.

We assessed the concordance among the four variant callers for each NGS platform. For our concordance analysis, variants identified with different read aligners were merged for each variant caller, and filtered out low confident variants below the quality score (QUAL) threshold of 20. For Illumina data sets, we observed ~92% of concordance among the variant calls by three variant callers (see GATK-HC ∩ Samtools ∩ Freebayes of Fig. 3A) based on the average score of data sets. In contrast to the previous studies which reported ~57%^16^ and ~70%^20^ of concordance levels among variant-calling pipelines for Illumina HiSeq2000 samples, our results showed substantially higher concordance. The inconsistent levels of concordance among these studies could be attributed to the version difference of software for aligners and callers, the differences in variant calling pipelines subjected to the analysis in different studies. Concordance levels among variant calling pipelines also varied across the data sets (82~97% overlap of called variants; see Supplementary Table 3). These results indicate that not only the variant calling pipelines but also the data sets affect concordance of the identified variants. Therefore, caution is advised in interpreting concordance levels based on a single data set. For Ion Proton data set, four callers showed 15.5% of overlap for the same quality score threshold (see GATK-HC ∩ Samtools ∩ Freebayes ∩ TVC of Fig. 3B). This low overlap among called variants is likely to originate from the high false positive rates for calling indel variants by Freebayes and Samtools (Supplementary Table 2).

### Variant callers are biased toward different types of SNP calling errors

SNP genotyping errors are generally divided into three classes: i) ignoring the reference allele (IR), ii) adding the reference allele (AR), and iii) other SNP calling errors. IR represents a homozygous SNP in the variant call, which is in fact a heterozygous SNP composed of one reference allele and one variant allele in the gold standard. Conversely, AR represents a heterozygous SNP composed of one reference allele and of one variant allele in the variant call, yet a homozygous SNP in the gold standard. The other class of SNP calling errors includes all other types of erroneous SNP calls regardless of the reference allele (see specific examples in Table 2). Among a total of 19,851 erroneous SNPs identified from the twelve data sets using the thirteen variant calling pipelines, 7,290 cases, 9,917 cases, and 2,644 cases were IR, AR, and the other types of SNP genotyping errors, respectively.

**Figure 4:**
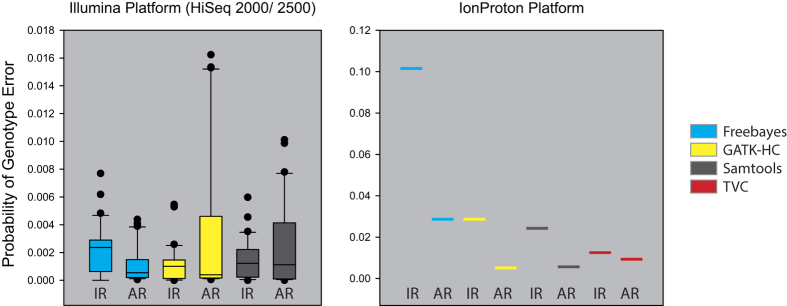
Probability of two types of SNP genotyping errors (IR and AR) for each caller as a function of different sequencing platforms. Probabilities of IR or AR for twelve Illumina data sets are summarized as box-and-whisker plots.

Probabilities of IR and AR for each of the four variant callers are summarized in Fig. 4. For Illumina platforms, Freebayes showed a higher probability of IR errors than AR errors. Conversely, both GATK-HC and Samtools showed a higher probability of AR errors than IR errors. These results suggest that Freebayes mainly prefers homozygous SNP calls, while GATK-HC and Samtools prefer heterozygous SNP calls of one reference allele and one variant allele for sequencing data on Illumina platforms. Therefore, for accurate variant calls with Illumina sequenced samples, caution is advised regarding homozygous SNP calls by Freebayes and heterozygous SNP calls by GATK-HC and Samtools.

For the Ion Proton platform, we observed bias toward IR errors for all four variant callers. Notably, the bias of Freebayes toward IR errors with Illumina data became more severe for Ion Proton data, and the directions of SNP calling error bias of GATK-HC and Samtools between IR and AR were all reversed relative to Illumina data. The increased probability of ignoring the reference allele in the Ion Proton data set can be attributable to the observed higher false positive rates by all variant callers over that seen for the Illumina data sets (see Fig. 2).

## Discussion

Revolutionary NGS technologies have remarkably decreased the cost of genome sequencing. This affordability of NGS allows the clinical application of WES or WGS to identify variants of personal genomes for practicing genomic medicine. During the past several years, many variant calling software tools have been developed. Variant identification for clinical genomics needs to be consistent and accurate, because false variant calls may lead to malpractice of genomic medicine, potentially resulting in enormous unnecessary personal and social cost. Therefore, understanding the overall performance and biases in false positive types of variant callers for each sequencing platform is a necessary task to advance genomic medicine. Nevertheless, a thorough performance comparison among variant calling methods has been difficult due to the lack of high-confident variant calls for benchmarking. Only recently has such benchmark variant data become available for a 1000 Genome Project individual, NA12878, by NIST GIAB consortium^19^. Therefore, we conducted a systematic comparison of different variant calling methods by using the benchmark variant calls.

In this study, we compared thirteen pipelines including popular variant calling software tools (GATK-HC, Samtools, Freebayes, and TVC) and popular read aligners (BWA-MEM, Bowtie2, Novoalign) against twelve sequence data sets for a single genome, NA12878. We employed precision-recall (PR) curves for benchmarking performance, because it is known to be more informative than another popular method, receiver operating characteristic (ROC) curves in benchmarking analysis for imbalanced data set, in which the negatives severely outnumber the positives^23^,^24^. When we measured the performance of variant callers using an area under the precision-recall curve (APR) for the SNP calls from Illumina data sets, the pipeline composed of BWA-MEM and Samtools showed the best performance, but Freebayes with any aligner showed an equally high performance for the SNP calls. For the SNPs from Ion Proton data set, Samtools outperformed all others, including TVC. For the indels from Illumina data sets, GATK-HC performed better than the other callers, regardless of the combined read aligner for the pipeline. However, we could not rule out the possibility that gold standard has some bias toward GATK-HC in indel calls, because the gold standard indel variants were made based on GATK-UnifiedGenotyper, GATK-HC and Cortex^25^.

Filtration of low quality calls (by quality score threshold of 20) can affect the overall performance of variant calling pipelines. We observed generally higher performance by pipelines with Freebayes than Samtools in SNP calling with filtered variant calls (Supplementary Figure 2). One possible explanation for the results is that there could be more false positives with low scores by Freebayes than by Samtools. Removal of variants with low score can increase the proportion of true positives of total called variants. These results suggest that Freebayes could be the better choice of variant caller if only high quality variants are considered for the study. Pipelines with GATK-HC have no changes by filtration in performance, because it reports variant whose quality score is higher than 30 only.

Another possible factor that influences variant calling performance is exome coverage. Using the analyzed eleven data sets for Illumina platform, which exhibit a wide range of exome coverage (43.6×–298.5×), we tested for correlation between exome coverage and performance of variant calling. We observed no significant correlation between exome coverage and APR (Supplementary Figure 3), indicating that variant calling performance is not significantly affected by this range of exome coverage. However, the tested Ion Proton data set has much lower exome coverage (<10×), and variant callers for the data set showed substantially lower performance. We examined the ratio between coverage of correctly called variants for heterozygous positions and those for homozygous positions, and found that the ratio for Ion Proton data is 0.79, whereas that for Illumina data is 1.63. These results indicate that low read depth of the Ion Proton data set decrease accuracy in variant calling, which led to the higher error rates.

Comparisons of variant calling pipelines on the GIAB gold standard variant set were conducted by two published studies: Cornish *et al.*^20^ and Highnam *et al.*^21^. To facilitate clinical genomics researchers in better comparing these studies with ours, we summarize the major differences in Supplementary Table 4 and show the performance of the thirteen pipelines as measured by PPV, rather than APR, in Supplementary Figure 4.

We analyzed concordance of the four callers for each sequencing platform and found high concordance for confident variants (QUAL 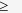
20) for Illumina data sets, but not for Ion Proton data set. The observed low concordance for Ion Proton data set could be attributed to the low exome coverage, which generated many false calls of indel variants by Samtools and Freebyaes (Supplementary Table 2). In addition, we observed that concordance levels for Illumina data sets vary from ~82% to ~97% (Supplementary Table 3). Therefore, any interpretation of the concordance results needs to consider not only differences in variant calling pipelines but also differences in attributes of data sets such as exome capture regions, exome coverage, and sequencing quality. We advise caution in interpreting results based on a single data set.

To further characterize variant callers, we also examined biases in SNP calling errors by each variant caller. We could divide the SNP calling errors into three classes: ignoring the reference allele (IR), adding the reference allele (AR), and an assortment of other minor cases regardless of the reference allele. We observed that Freebayes has higher preference in generating IR, whereas Samtools and GATK-HC more frequently generate AR. These results suggest that, for accurate variant calls, some caution is merited regarding homozygous SNP calls using Freebayes and heterozygous SNP calls by GATK-HC and Samtools.

Taken together, our recommendation is the use of BWA-MEM and Samtools pipeline for SNP calls and BWA-MEM and GATK-HC pipeline for indel calls. For Ion Proton data, Samtools appears the best current approach for both SNPs and indels. However, users of Samtools may need to be more cautious about heterozygous SNPs, because it tends to add reference alleles. We conclude that results from our study will provide practical and comprehensive guidance to more accurate and consistent variant identification, ultimately leading to the clinical-grade personal variant information for genomic medicine.

## Materials and Methods

### Data sets

One individual (NA12878) from the 1000 genome project was sequenced in parallel with the HiSeq2000, HiSeq2500 and Ion Proton platforms, and annotated as the gold standard reference benchmark^19^. Twelve sequenced read data sets for NA12878 were downloaded from public databases: eleven Illumina data sets from Sequence Read Archive (SRA) of National Center of Biotechnology Information (NCBI)^22^ and one Ion Proton data set from GIAB FTP site of National Institute of Standards and Technology (NIST)^19^ (Table 1). We excluded data sets realigned using GATK IndelRealigner, because the standard procedure of the Freebayes does not adopt the GATK realignment steps. To avoid biased results by NGS platform, we used a similar number of data sets sequenced by HiSeq2000 (seven sets) and HiSeq2500 (four sets). However, there was only one NA12878 sequence data set for Ion Proton available from the public database, as of this study.

The sequence reads downloaded from SRA were converted to bam files of the binary version of sequenced alignment/map files (known as sam files) or fastq files using sratools-2.4.2 depending on their alignment status. For Illumina data sets, we mapped them to the GRCh37 reference genome through BWA-MEM-0.7.10, Bowtie2-2.2.25, and Novoalign-3.02.12. The downloaded data set for Ion Proton was pre-mapped to the GRCh37 by Ion Proton read aligner, Tmap. To remove duplicates in each bam files, we used MarkDuplicate in Picard-bf40841. To estimate exome and genome coverage of each sample, we used the bedtool2 program^26^ and the UCSC knownGene bed file^27^ that has only protein coding exon information.

### Identifying variants using four variant calling methods

We ran four variant calling methods: (1) Genome Analysis Tool Kit-3.3.0 HaplotypeCaller (GATK-HC), (2) Samtools-1.1, (3) Freebayes-0.9.18, and (4) Torrent Variant Caller (TVC)-4.2.3. For calling variants by GATK-HC and Samtools, we ran ReorderSam in Picard, IndelRealigner and BaseRecalibrator in GATK for all samples, by following their own best practice procedures. We also used the default parameters or suggested input files, such as the most recent dbSNP vcf file, a HapMap genotype file and OMNI 2.5 genotype vcf file. For calling variants by Freebayes, we used mark-duplicated bam files, described in the previous section without any additional process. For the genomic sequence data by Ion Proton, we also ran its own variant calling method, TVC-4.2.3 standalone version. As the last step of calling variants, we regularized the representation of the variants by using vcfallelicprimitive module in Vcflib, because the same variant could be represented in different ways by different methods. All methods were tested on Linux CentOS 6.

### The gold standard genotype data

For benchmarking variant callers, the highly confident heterozygous and homozygous variant calls provided by NIST GIAB consortium (http://www.genomeinabottle.org) on the only exon regions without known structural variants were used as the gold standard variants set. We used NIST_RTG_PlatGen_merged_highconfidence_v0.2_Allannotate.vcf file, which was regularized by vcfallelicprimitive module in Vcflib. Also we used high confident homozygous reference alleles as a negative set which is provided by GIAB as a bed file, union13callableMQonlymerged_addcert_nouncert_excludesimplerep_excludesegdups_excludedecoy_excludeRepSeqSTRs_noCNVs_v2.19_2mindatasets_5minYesNoRatio_AddRTGPlatGenConf_filtNISTclustergt9_RemNISTfilt_RemPartComp_RemRep_RemPartComp_v0.2.bed. Then, the variants within intron and non-protein coding regions were filtered out against exome capture regions of each data set of hg19 protein coding exon regions annotated by UCSC genome browser^27^, SeqCap EZ Human Exome Lib v3.0, SeqCap EZ Exome SeqCap v2, GeneDx_Sureselectv4, and SureSelect v2 for whole exome sequence data sets, SRR16111XX series data sets, SRR292250, SRR515199, and SRR098401, respectively (Table 1). We also used gold standard SNPs and indels in intersection regions of the high confident region and exome capture region. A total of SNPs and indels of NA12878 that were used as benchmark variant data in this study are shown in Supplementary Tables 1 and 2.

### Performance measure of variant calling pipelines

To assess the performance of variant calling pipelines, we used precision-recall curves. We defined true positive (TP), true negative (TN), false positive (FP), and false negative (FN) variants as follows:

TP: variants called by a variant caller as the same genotype as the gold standard data

TN: reference alleles in high confident regions other than gold standard variants

FP: variants called by a variant caller but not in the gold standard variant set.

FN: gold standard variants that were not called by a variant caller

Precision: TP/(TP+FP)

Recall: TP/(TP+FN)

We drew PR curves for SNPs and indels for each of the thirteen variant calling pipelines, separately, and then calculated area under the precision-recall curve (APR) as a summary score. APR ranges from zero to one, where a score of one indicates perfect variant calls.

## Additional Information

**How to cite this article**: Hwang, S. *et al.* Systematic comparison of variant calling pipelines using gold standard personal exome variants. *Sci. Rep.* **5**, 17875; doi: 10.1038/srep17875 (2015).

## Supplementary Material

Supplementary Information

## Figures and Tables

**Table 1:** Summary of data sets used in this study.

Plaform	Accession	Exome Capture	WGS/WES	Exome coverage
HiSeq2000	SRR1611178	SeqCap EZ Human Exome Lib v3.0	WES	79.93×
HiSeq2000	SRR1611179	SeqCap EZ Human Exome Lib v3.0	WES	79.84×
HiSeq2000	SRR292250	SeqCap EZ Exome SeqCap v2	WES	116.06×
HiSeq2000	SRR515199	SureSelect v4	WES	298.45×
HiSeq2000	SRR098401	SureSelect v2	WES	116.84×
HiSeq2500	SRR1611183	SeqCap EZ Human Exome Lib v3.0	WES	129.94×
HiSeq2500	SRR1611184	SeqCap EZ Human Exome Lib v3.0	WES	111.90×
HiSeq2000	ERR194147	UCSC Known gene	WGS	45.68×
HiSeq2000	SRX485062	UCSC Known gene	WGS	56.60×
HiSeq2500	SRX515284	UCSC Known gene	WGS	56.87×
HiSeq2500	SRX516752	UCSC Known gene	WGS	43.61×
IonProton	NA12878_combine	UCSC Known gene	WGS	9.87×

**Table 2:** Examples of SNP calling errors.

	In a variant call set	In gold standard set
Ignoring the reference allele (IR)	AA	RA
Adding the reference allele (AR)	RA	AA
Other SNP FP calls	AA	AB
	AB	AA
	RA	RB

R, reference allele; A, B, alternative SNP allele
