# Supplementary material for: Systematic comparison of variant calling pipelines using gold standard personal exome variants
Source: Sci Rep. 2015 Dec 7;5:17875. doi: 10.1038/srep17875 (PMC4671096; doi:10.1038/srep17875)
Supplement: Supplementary Information [file srep17875-s1.pdf]

Supplementary Online Information

Systematic comparison of variant calling pipelines using gold standard personal exome variants

Sohyun Hwang, Eiru Kim, Insuk Lee and Edward M Marcotte

Supplementary Figure 1. Precision-Recall curves for calling SNPs and indels from twelve NA12878 samples using thirteen pipelines.

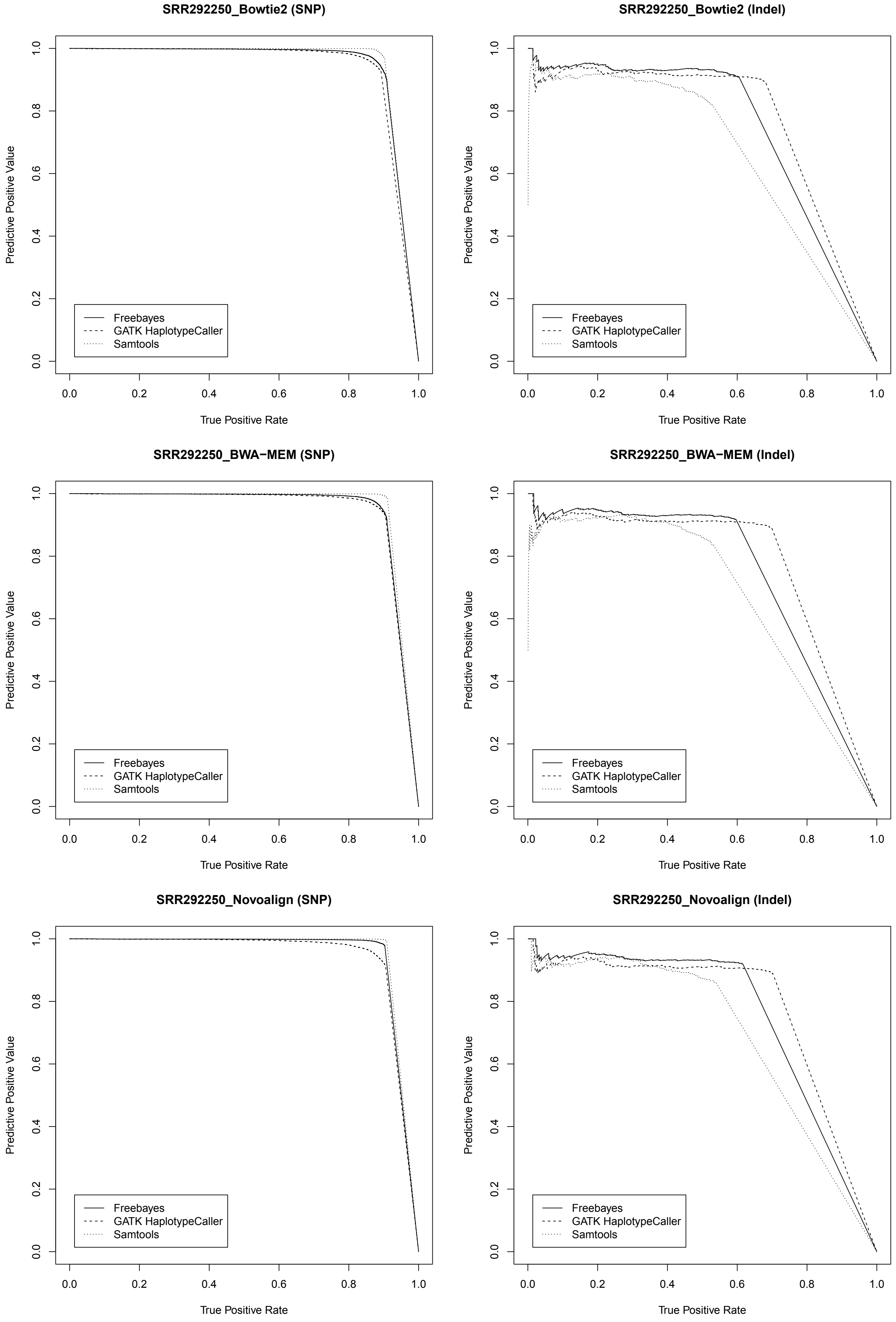

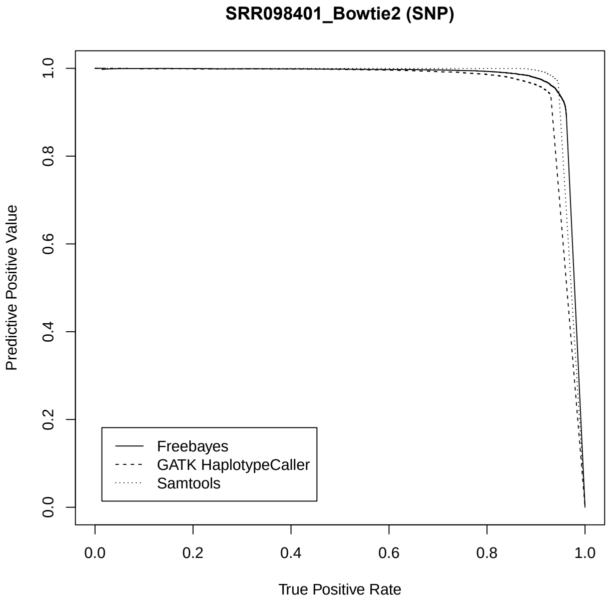
SRR098401_Bowtie2 (SNP)
Predictive Positive Value
True Positive Rate
0.0
0.2
0.4
0.6
0.8
1.0
Freebayes
GATK HaplotypeCaller
Samtools

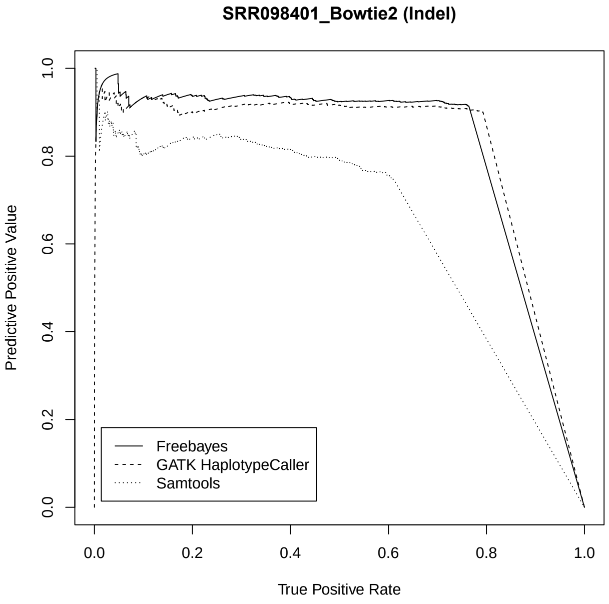
SRR098401_Bowtie2 (Indel)
Predictive Positive Value
True Positive Rate
0.0
0.2
0.4
0.6
0.8
1.0
Freebayes
GATK HaplotypeCaller
Samtools

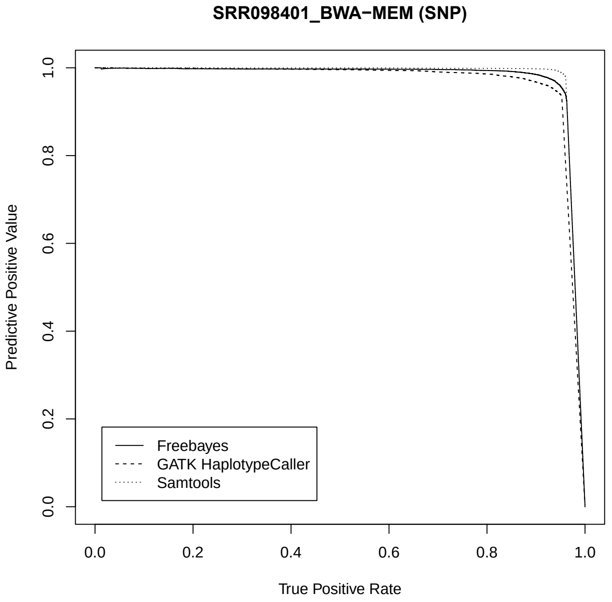
SRR098401_BWA−MEM (SNP)
Predictive Positive Value
True Positive Rate
0.0
0.2
0.4
0.6
0.8
1.0
Freebayes
GATK HaplotypeCaller
Samtools

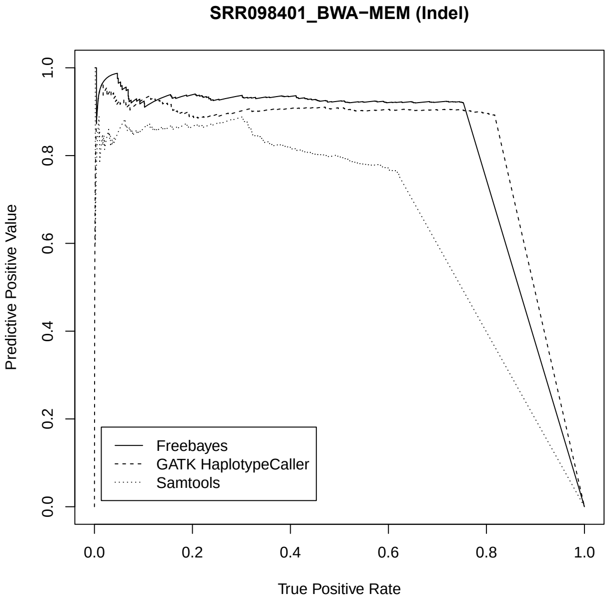
SRR098401_BWA−MEM (Indel)
Predictive Positive Value
True Positive Rate
0.0
0.2
0.4
0.6
0.8
1.0
Freebayes
GATK HaplotypeCaller
Samtools

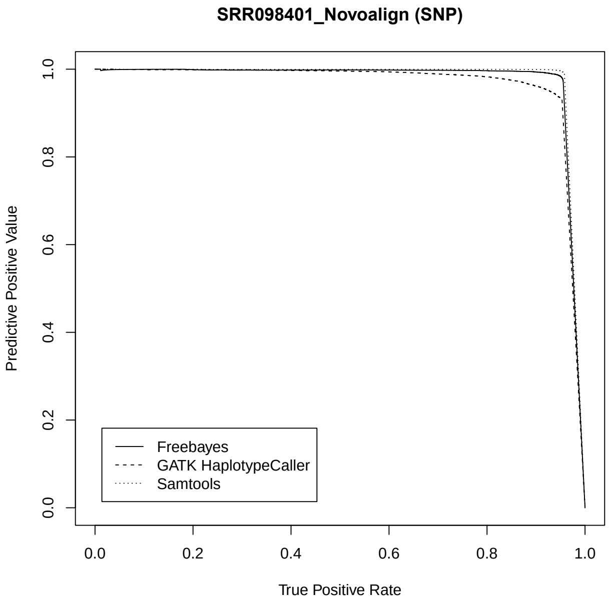
SRR098401_Novoalign (SNP)
Predictive Positive Value
True Positive Rate
0.0
0.2
0.4
0.6
0.8
1.0
Freebayes
GATK HaplotypeCaller
Samtools

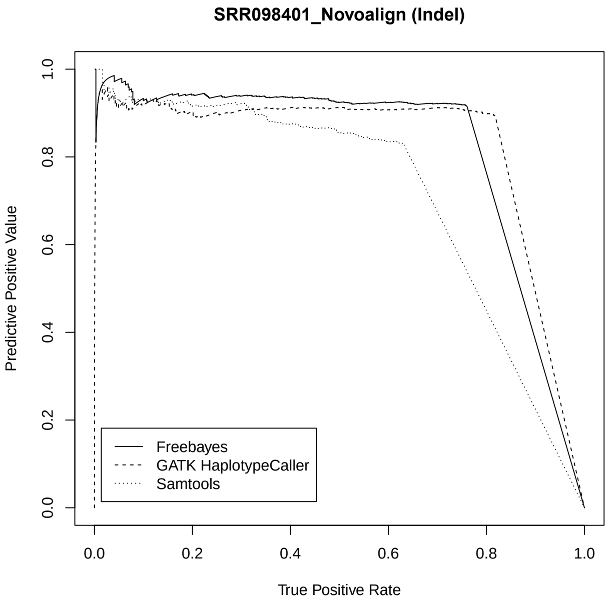
SRR098401_Novoalign (Indel)
Predictive Positive Value
True Positive Rate
0.0
0.2
0.4
0.6
0.8
1.0
Freebayes
GATK HaplotypeCaller
Samtools

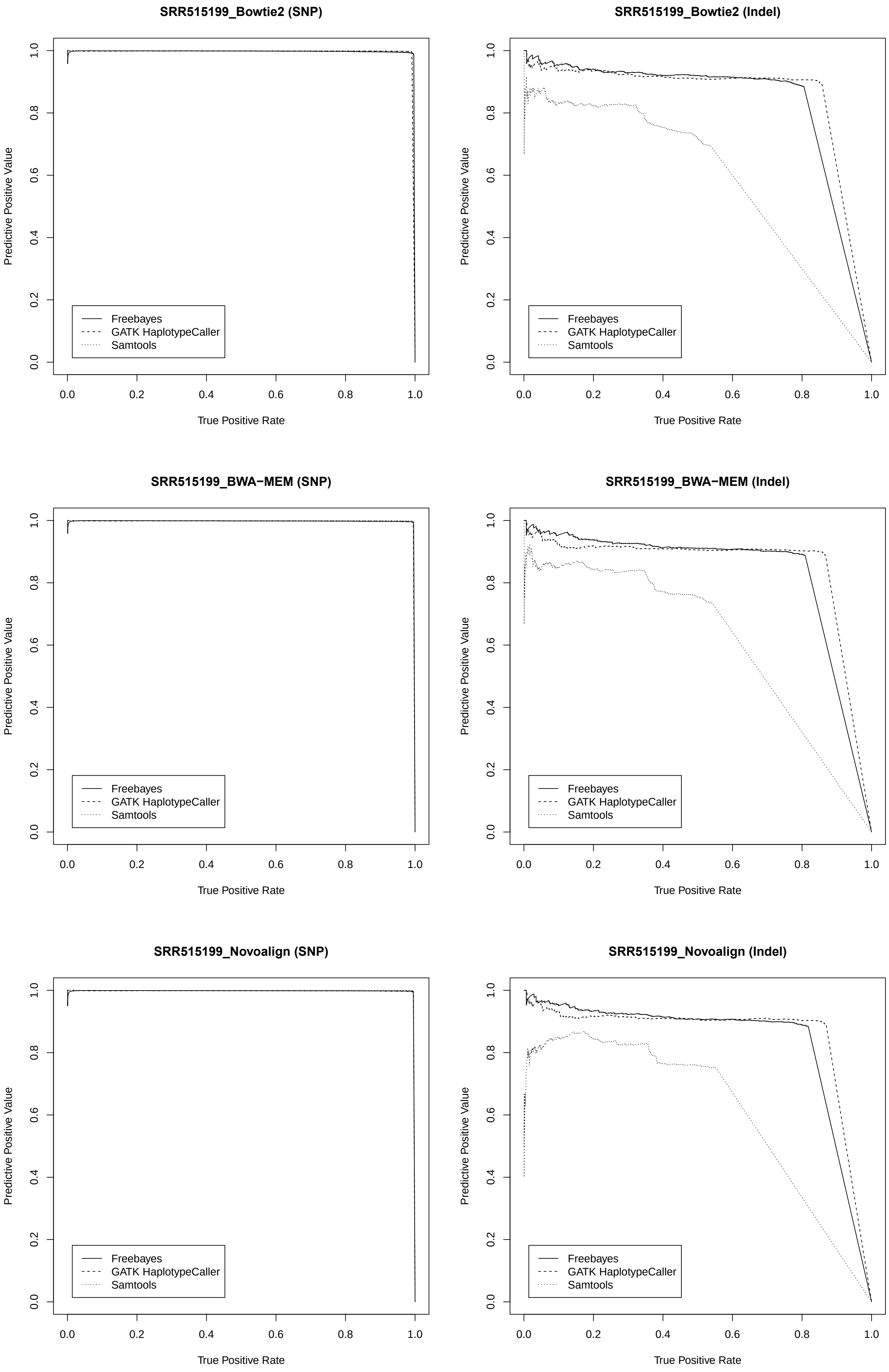

SRR515199_Bowtie2 (SNP)
SRR515199_Bowtie2 (Indel)
SRR515199_BWA−MEM (SNP)
SRR515199_BWA−MEM (Indel)
SRR515199_Novoalign (SNP)
SRR515199_Novoalign (Indel)
Predictive Positive Value
True Positive Rate
0.0
0.2
0.4
0.6
0.8
1.0
Freebayes
GATK HaplotypeCaller
Samtools

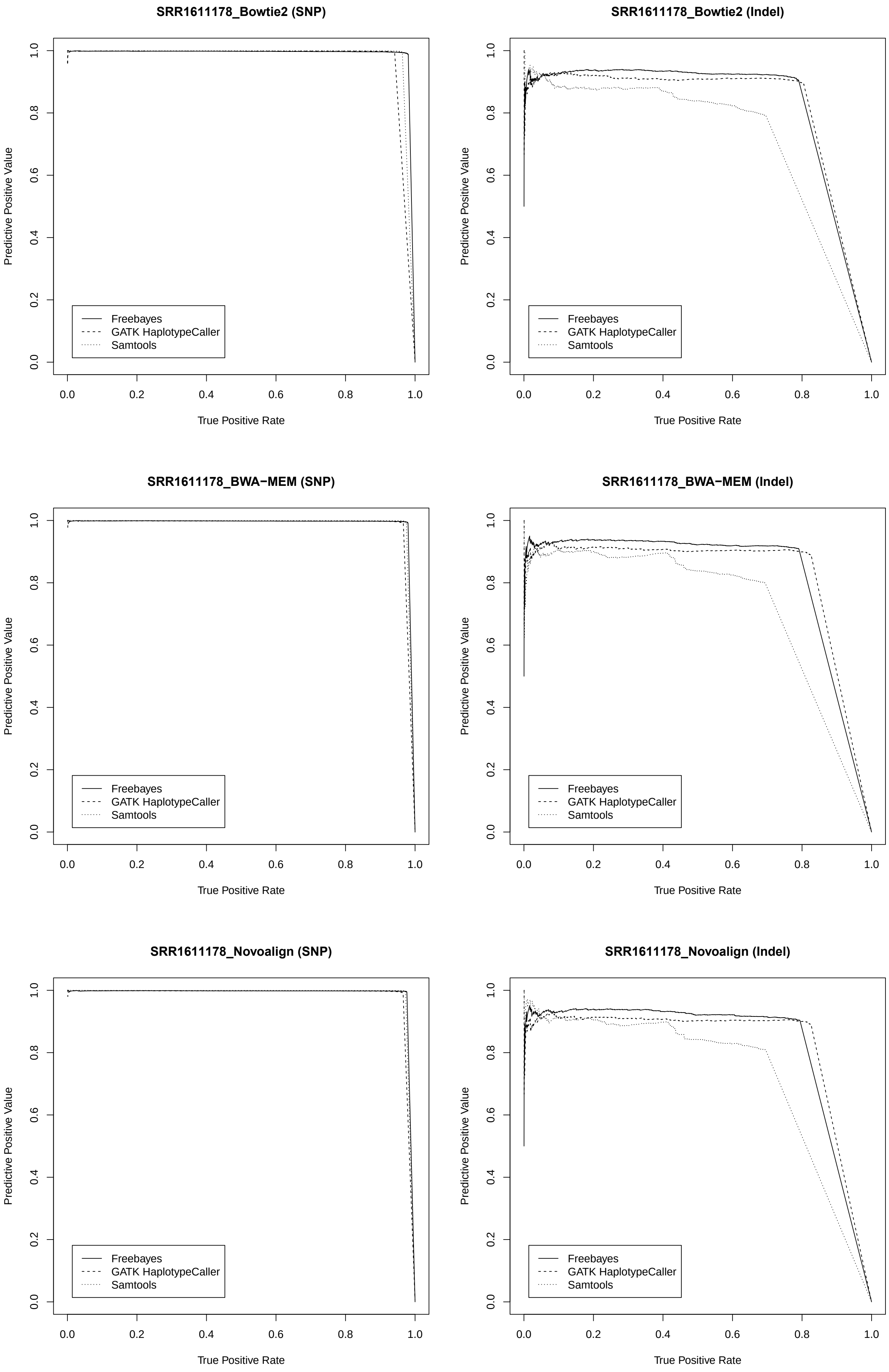
SRR1611178_Bowtie2 (SNP)
SRR1611178_Bowtie2 (Indel)
SRR1611178_BWA−MEM (SNP)
SRR1611178_BWA−MEM (Indel)
SRR1611178_Novoalign (SNP)
SRR1611178_Novoalign (Indel)
Predictive Positive Value
True Positive Rate
0.0
0.2
0.4
0.6
0.8
1.0
Freebayes
GATK HaplotypeCaller
Samtools

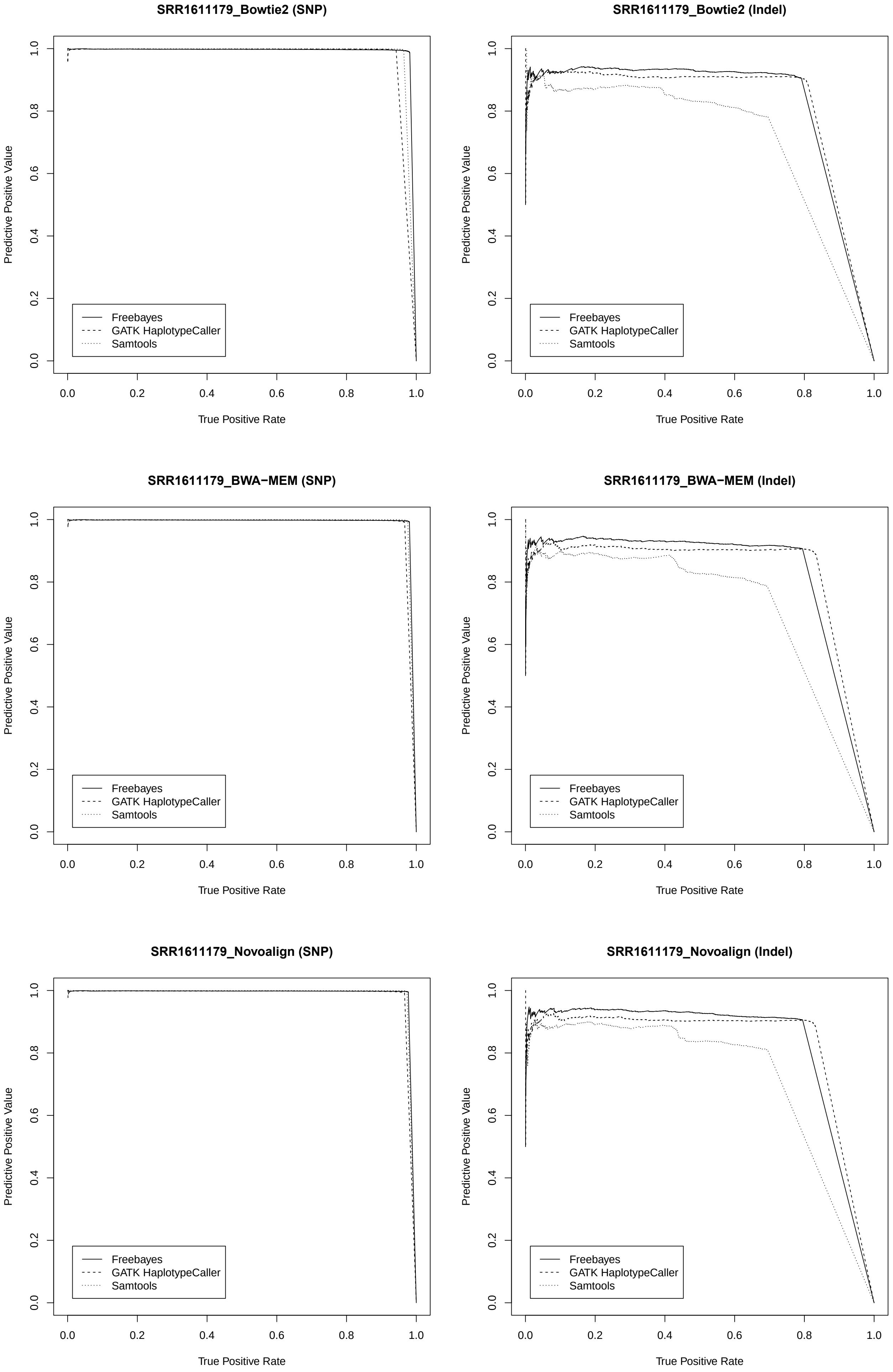
SRR1611179_Bowtie2 (SNP)
SRR1611179_Bowtie2 (Indel)
SRR1611179_BWA−MEM (SNP)
SRR1611179_BWA−MEM (Indel)
SRR1611179_Novoalign (SNP)
SRR1611179_Novoalign (Indel)
Predictive Positive Value
True Positive Rate
0.0
0.2
0.4
0.6
0.8
1.0
Freebayes
GATK HaplotypeCaller
Samtools

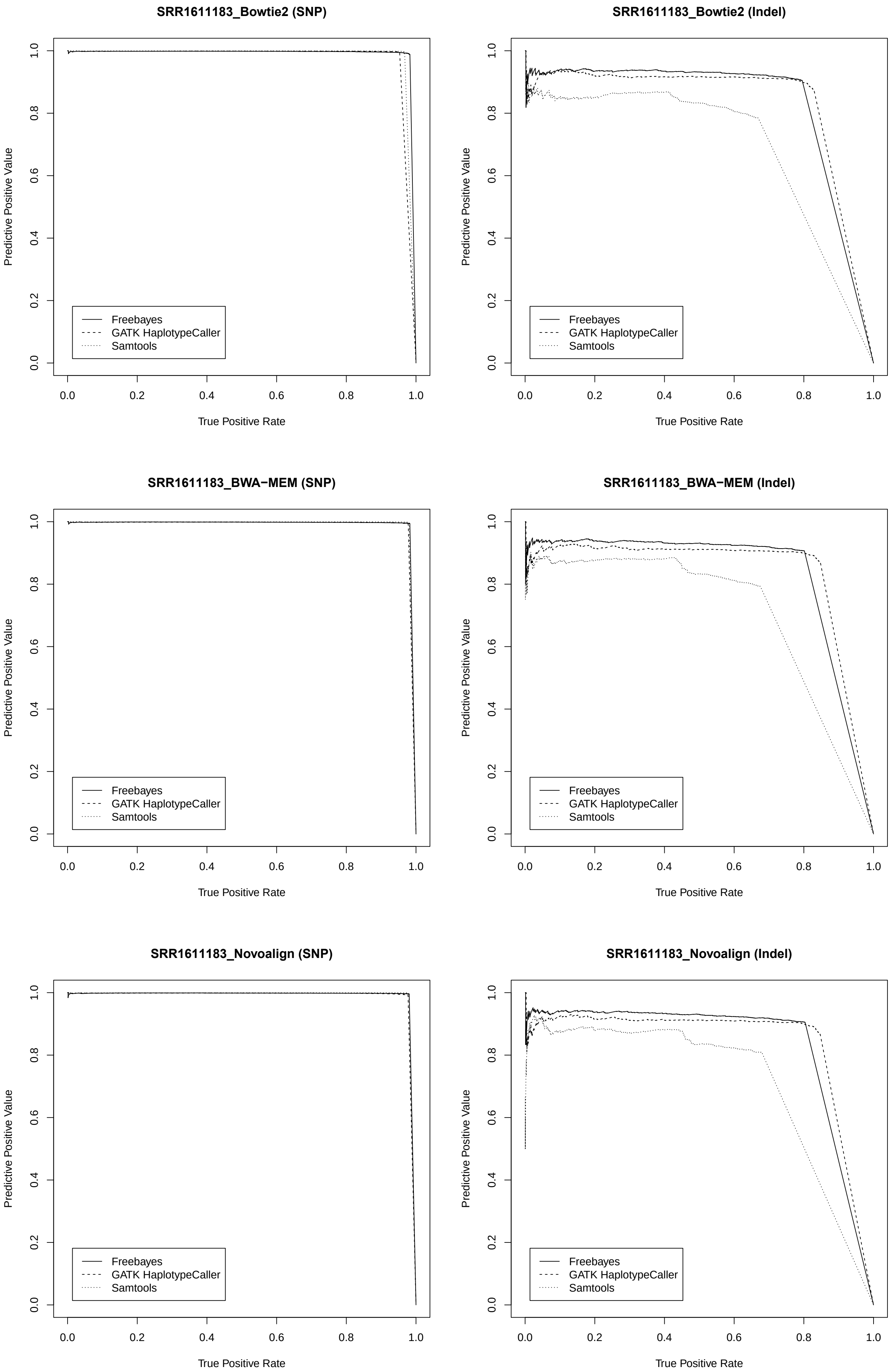
SRR1611183_Bowtie2 (SNP)
SRR1611183_Bowtie2 (Indel)
SRR1611183_BWA−MEM (SNP)
SRR1611183_BWA−MEM (Indel)
SRR1611183_Novoalign (SNP)
SRR1611183_Novoalign (Indel)
Predictive Positive Value
True Positive Rate
0.0
0.2
0.4
0.6
0.8
1.0
Freebayes
GATK HaplotypeCaller
Samtools

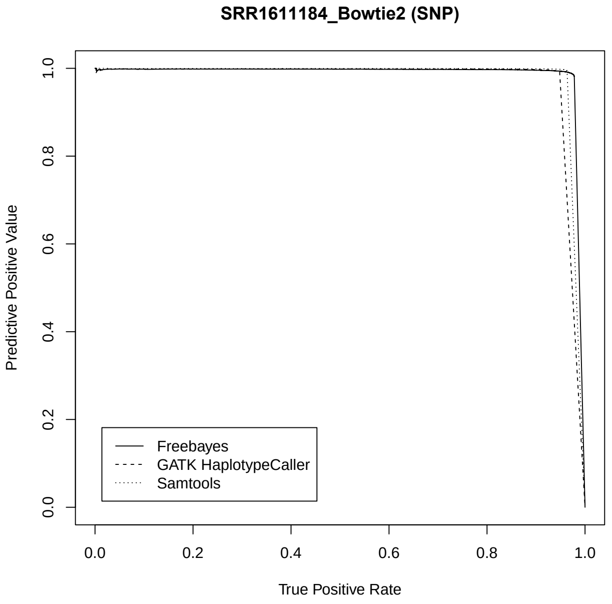
SRR1611184_Bowtie2 (SNP)
Predictive Positive Value
True Positive Rate
Freebayes
GATK HaplotypeCaller
Samtools

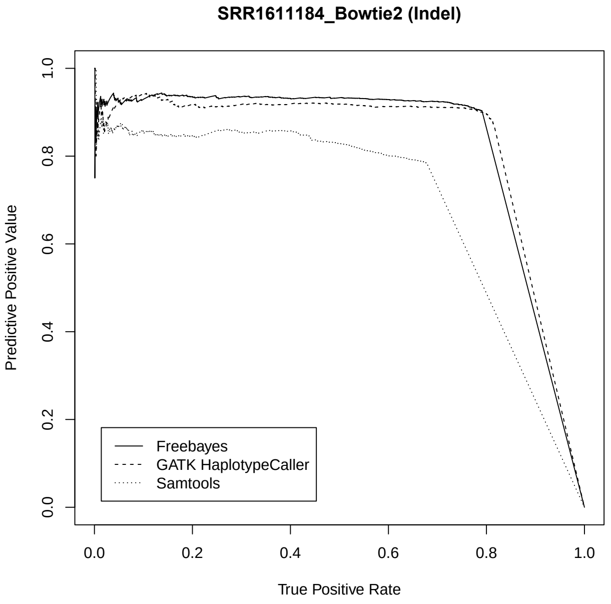
SRR1611184_Bowtie2 (Indel)
Predictive Positive Value
True Positive Rate
Freebayes
GATK HaplotypeCaller
Samtools

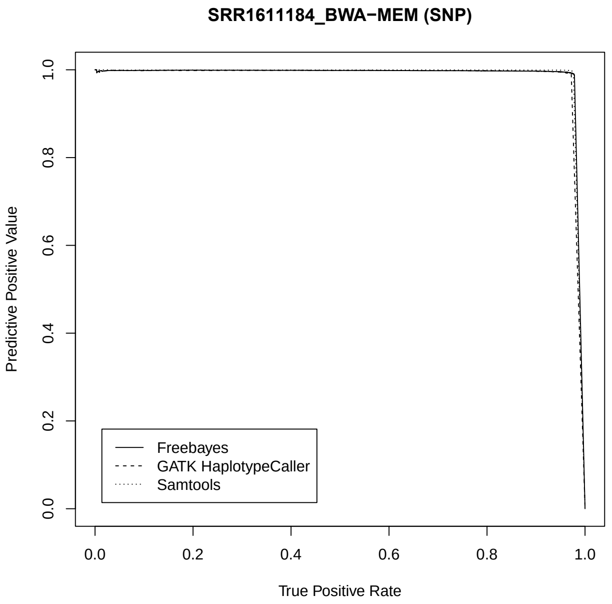
SRR1611184_BWA−MEM (SNP)
Predictive Positive Value
True Positive Rate
Freebayes
GATK HaplotypeCaller
Samtools

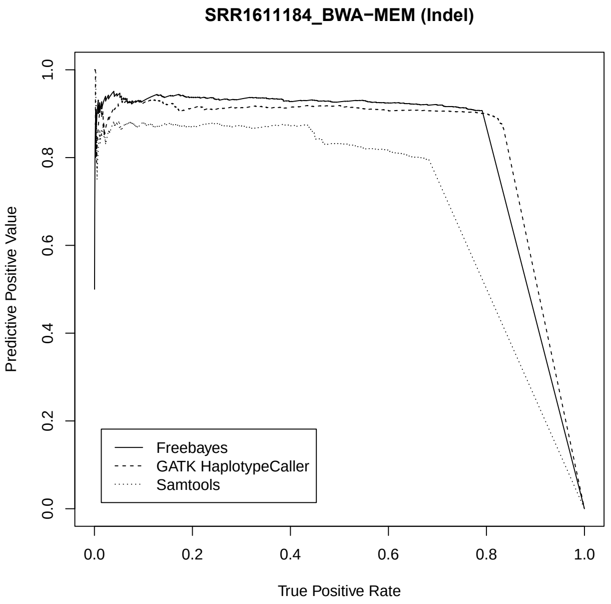
SRR1611184_BWA−MEM (Indel)
Predictive Positive Value
True Positive Rate
Freebayes
GATK HaplotypeCaller
Samtools

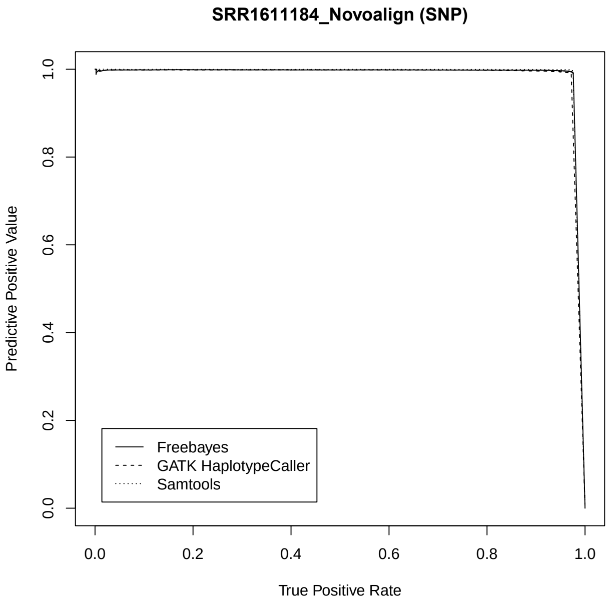
SRR1611184_Novoalign (SNP)
Predictive Positive Value
True Positive Rate
Freebayes
GATK HaplotypeCaller
Samtools

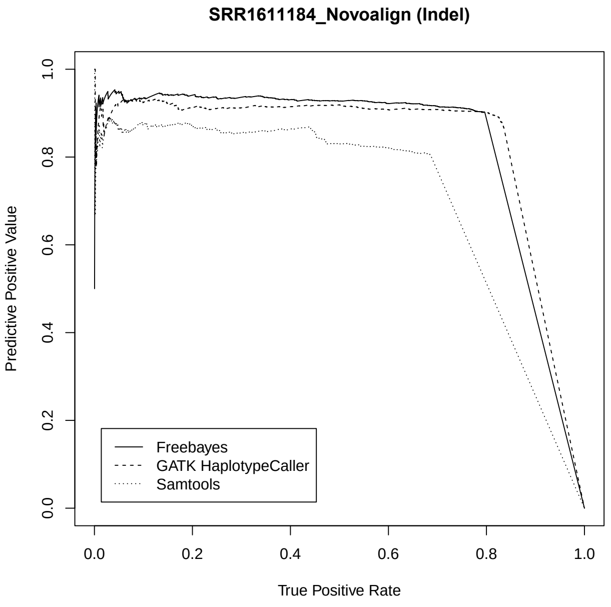
SRR1611184_Novoalign (Indel)
Predictive Positive Value
True Positive Rate
Freebayes
GATK HaplotypeCaller
Samtools

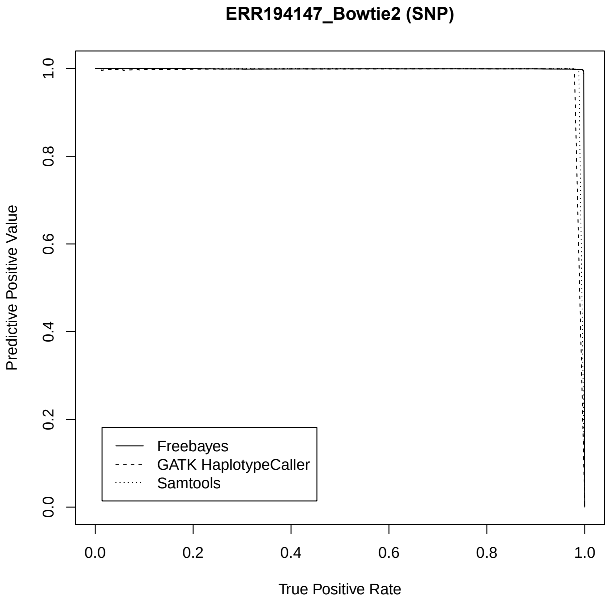
ERR194147_Bowtie2 (SNP)
Predictive Positive Value
True Positive Rate
0.0
0.2
0.4
0.6
0.8
1.0
Freebayes
GATK HaplotypeCaller
Samtools

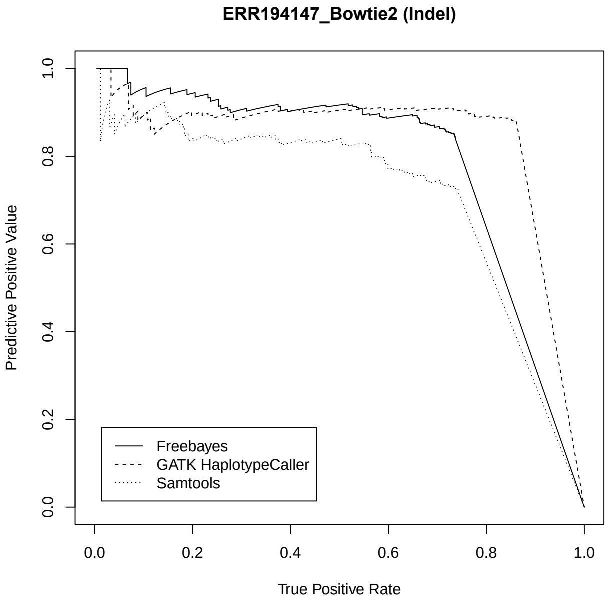
ERR194147_Bowtie2 (Indel)
Predictive Positive Value
True Positive Rate
0.0
0.2
0.4
0.6
0.8
1.0
Freebayes
GATK HaplotypeCaller
Samtools

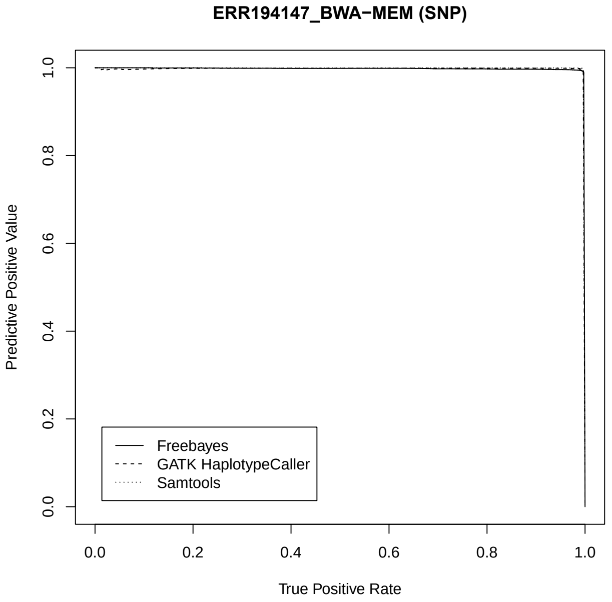
ERR194147_BWA−MEM (SNP)
Predictive Positive Value
True Positive Rate
0.0
0.2
0.4
0.6
0.8
1.0
Freebayes
GATK HaplotypeCaller
Samtools

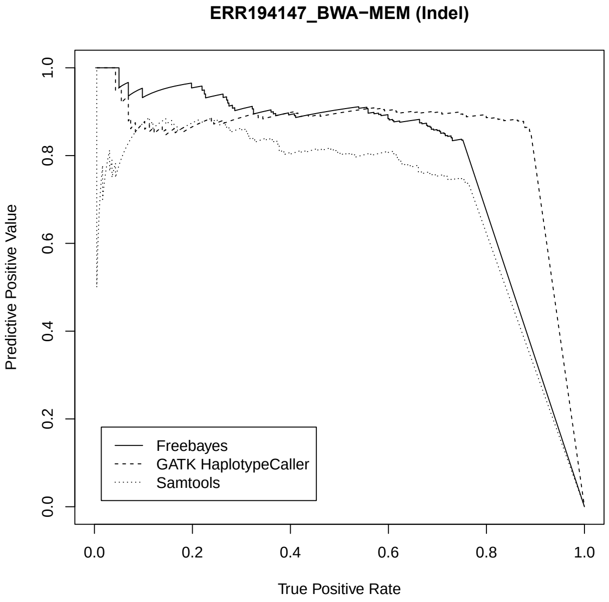
ERR194147_BWA−MEM (Indel)
Predictive Positive Value
True Positive Rate
0.0
0.2
0.4
0.6
0.8
1.0
Freebayes
GATK HaplotypeCaller
Samtools

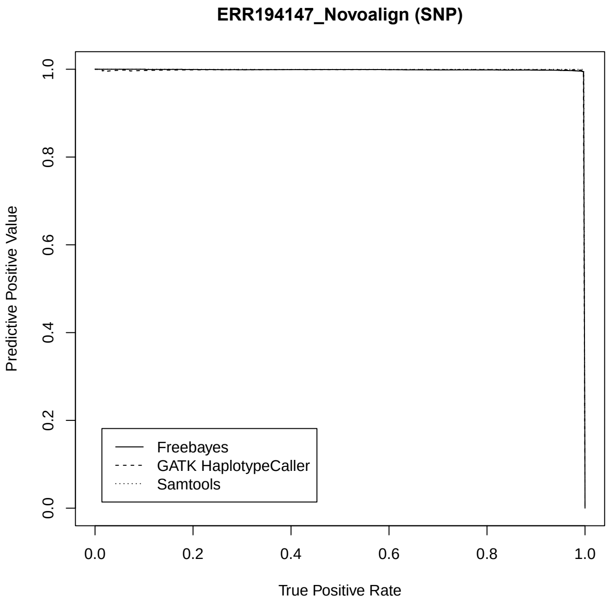
ERR194147_Novoalign (SNP)
Predictive Positive Value
True Positive Rate
0.0
0.2
0.4
0.6
0.8
1.0
Freebayes
GATK HaplotypeCaller
Samtools

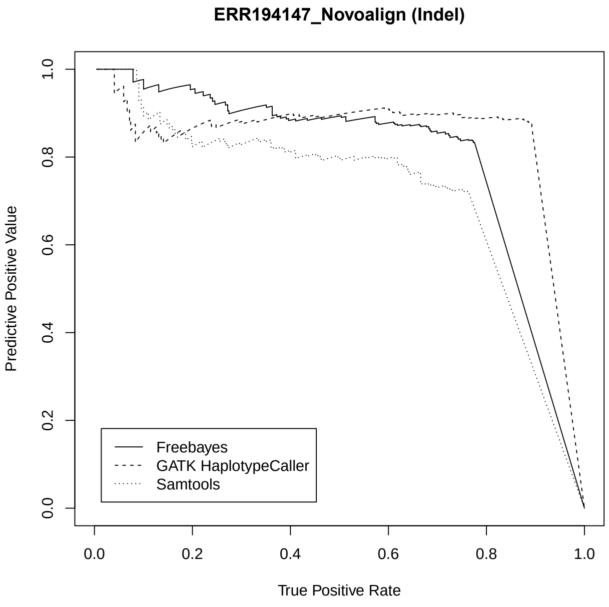
ERR194147_Novoalign (Indel)
Predictive Positive Value
True Positive Rate
0.0
0.2
0.4
0.6
0.8
1.0
Freebayes
GATK HaplotypeCaller
Samtools

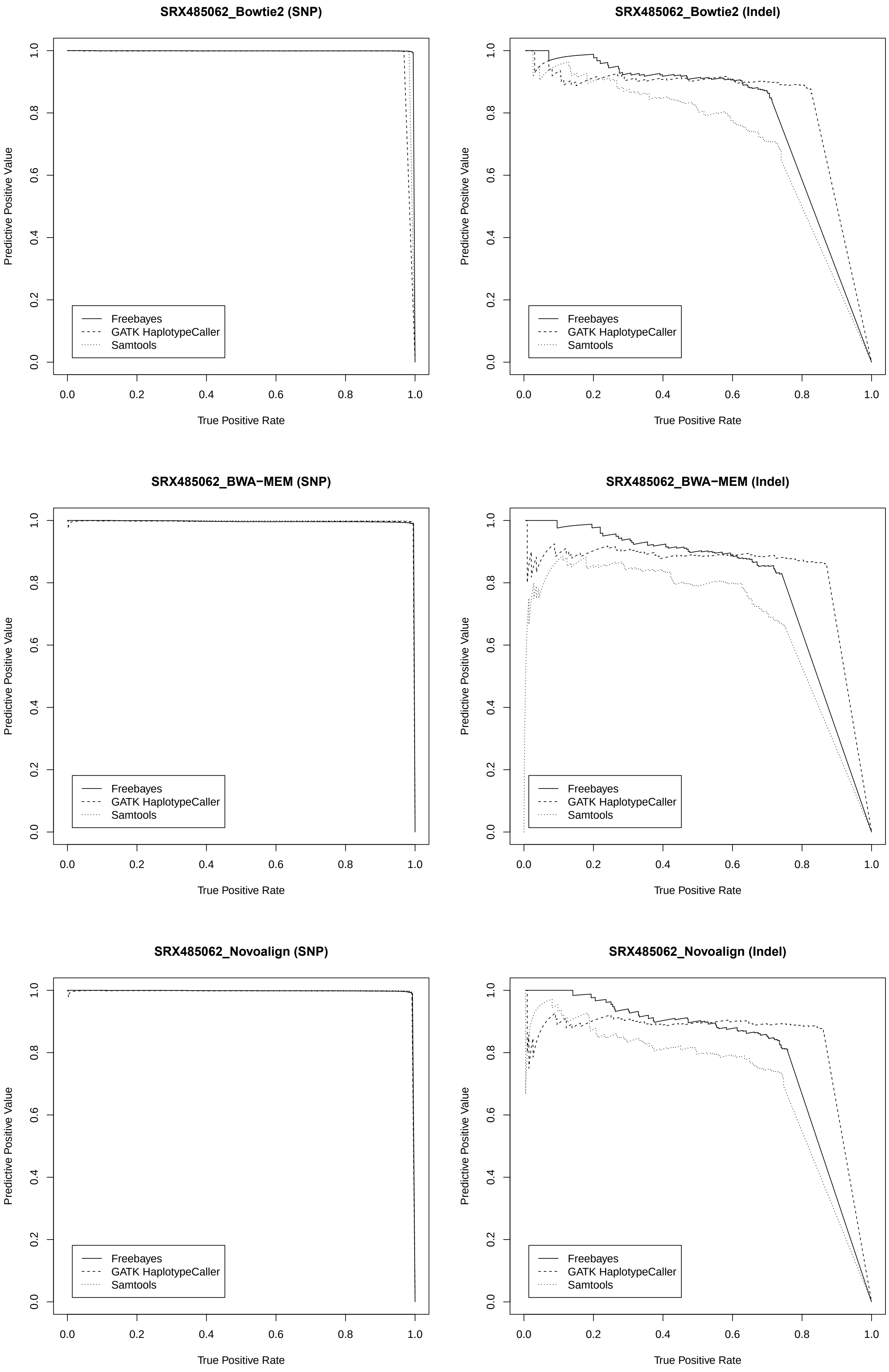
SRX485062_Bowtie2 (SNP)
SRX485062_Bowtie2 (Indel)
SRX485062_BWA−MEM (SNP)
SRX485062_BWA−MEM (Indel)
SRX485062_Novoalign (SNP)
SRX485062_Novoalign (Indel)
Predictive Positive Value
True Positive Rate
0.0
0.2
0.4
0.6
0.8
1.0
Freebayes
GATK HaplotypeCaller
Samtools

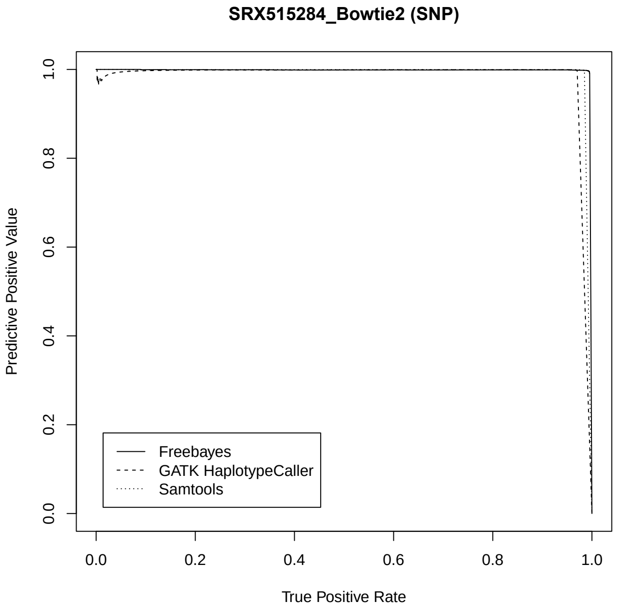
SRX515284_Bowtie2 (SNP)
Predictive Positive Value
True Positive Rate
0.0
0.2
0.4
0.6
0.8
1.0
Freebayes
GATK HaplotypeCaller
Samtools

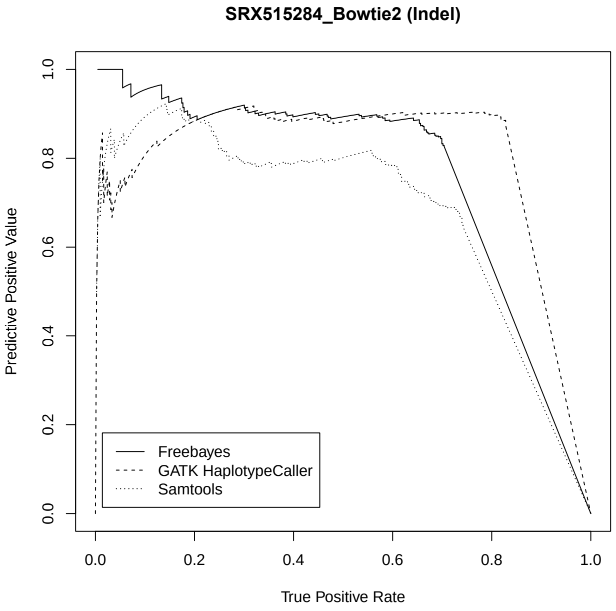
SRX515284_Bowtie2 (Indel)
Predictive Positive Value
True Positive Rate
0.0
0.2
0.4
0.6
0.8
1.0
Freebayes
GATK HaplotypeCaller
Samtools

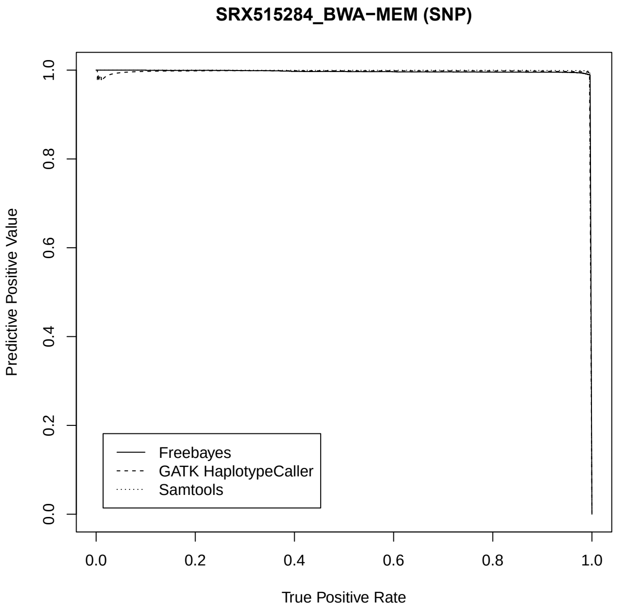
SRX515284_BWA−MEM (SNP)
Predictive Positive Value
True Positive Rate
0.0
0.2
0.4
0.6
0.8
1.0
Freebayes
GATK HaplotypeCaller
Samtools

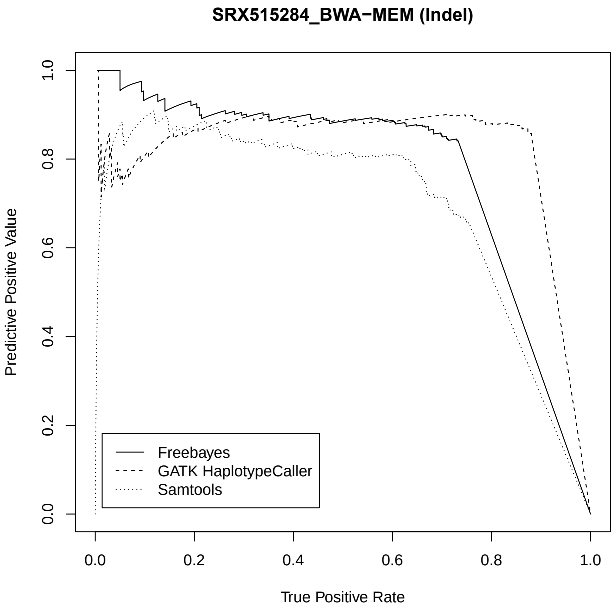
SRX515284_BWA−MEM (Indel)
Predictive Positive Value
True Positive Rate
0.0
0.2
0.4
0.6
0.8
1.0
Freebayes
GATK HaplotypeCaller
Samtools

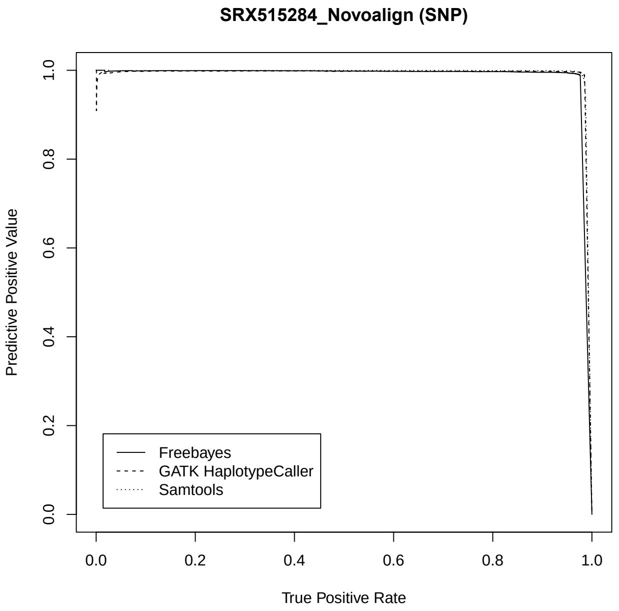
SRX515284_Novoalign (SNP)
Predictive Positive Value
True Positive Rate
0.0
0.2
0.4
0.6
0.8
1.0
Freebayes
GATK HaplotypeCaller
Samtools

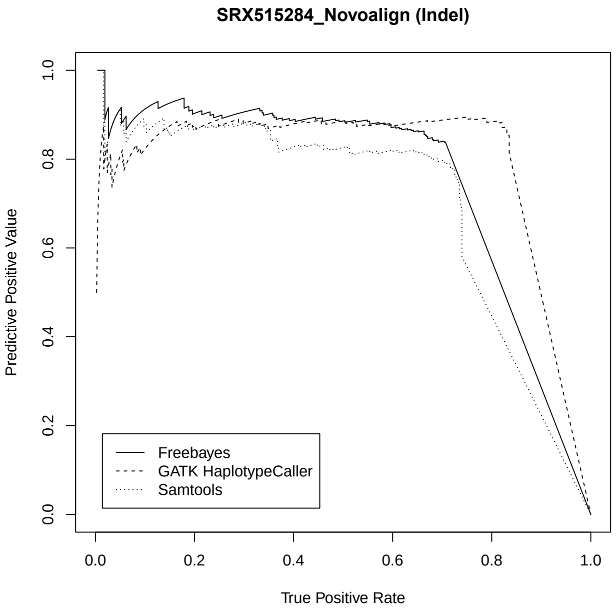
SRX515284_Novoalign (Indel)
Predictive Positive Value
True Positive Rate
0.0
0.2
0.4
0.6
0.8
1.0
Freebayes
GATK HaplotypeCaller
Samtools

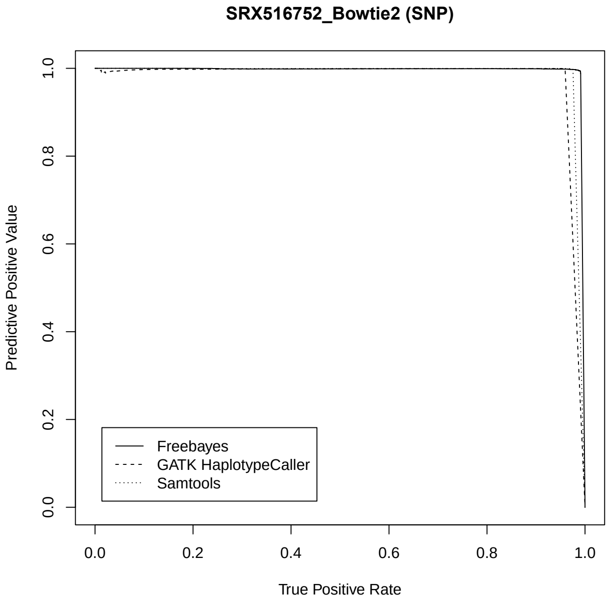
SRX516752_Bowtie2 (SNP)
Predictive Positive Value
True Positive Rate
0.0
0.2
0.4
0.6
0.8
1.0
Freebayes
GATK HaplotypeCaller
Samtools

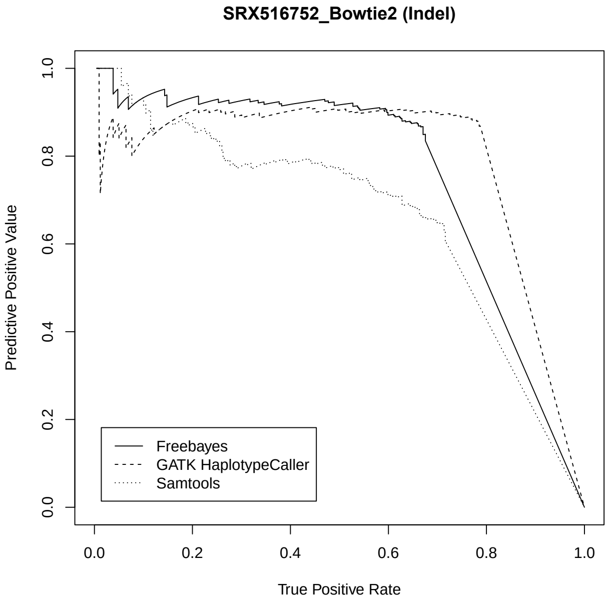
SRX516752_Bowtie2 (Indel)
Predictive Positive Value
True Positive Rate
0.0
0.2
0.4
0.6
0.8
1.0
Freebayes
GATK HaplotypeCaller
Samtools

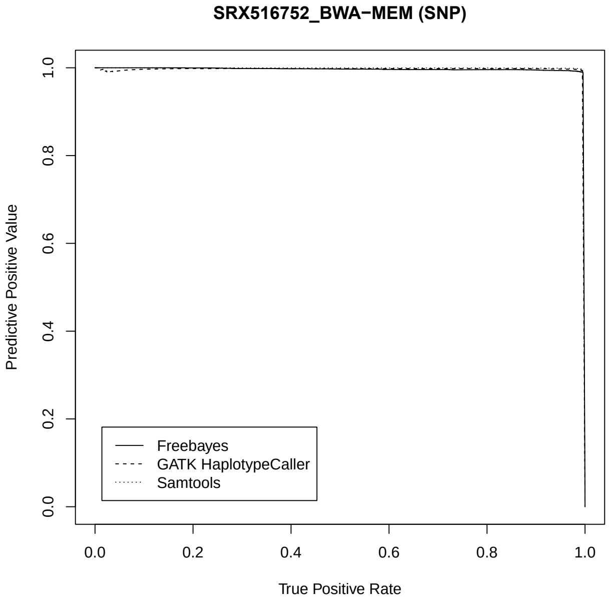
SRX516752_BWA−MEM (SNP)
Predictive Positive Value
True Positive Rate
0.0
0.2
0.4
0.6
0.8
1.0
Freebayes
GATK HaplotypeCaller
Samtools

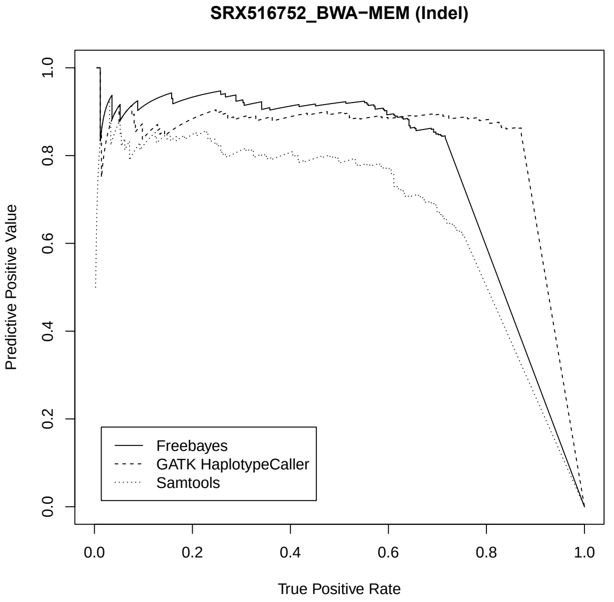
SRX516752_BWA−MEM (Indel)
Predictive Positive Value
True Positive Rate
0.0
0.2
0.4
0.6
0.8
1.0
Freebayes
GATK HaplotypeCaller
Samtools

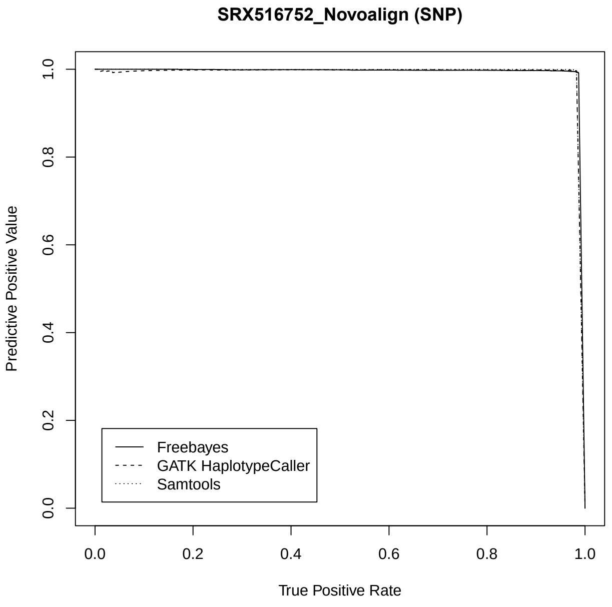
SRX516752_Novoalign (SNP)
Predictive Positive Value
True Positive Rate
0.0
0.2
0.4
0.6
0.8
1.0
Freebayes
GATK HaplotypeCaller
Samtools

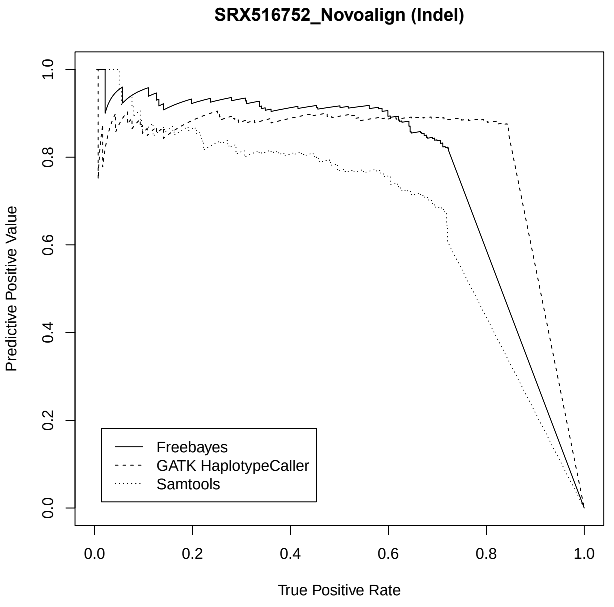
SRX516752_Novoalign (Indel)
Predictive Positive Value
True Positive Rate
0.0
0.2
0.4
0.6
0.8
1.0
Freebayes
GATK HaplotypeCaller
Samtools

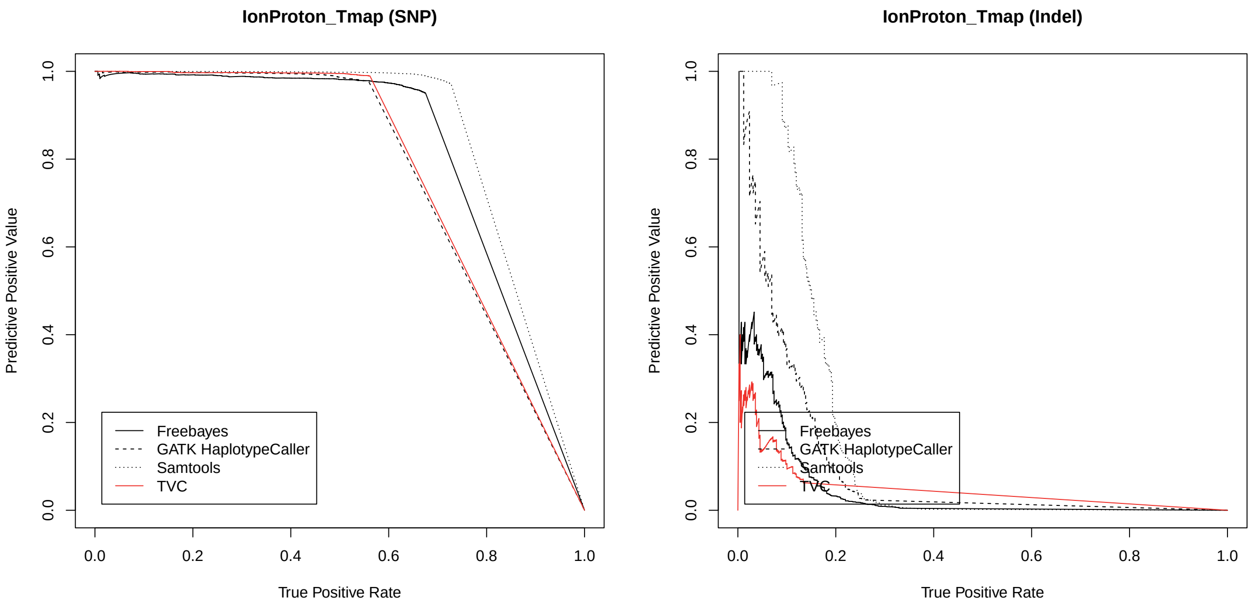
IonProton_Tmap (SNP)
IonProton_Tmap (Indel)
Predictive Positive Value
True Positive Rate
0.0
0.2
0.4
0.6
0.8
1.0
Freebayes
GATK HaplotypeCaller
Samtools
TVC

Supplementary Figure 2. Summary of variant calling performances by thirteen pipelines, with filtration of low scored variants. Performances were measured for (A) SNPs and (B) indels with score higher than 20 only.

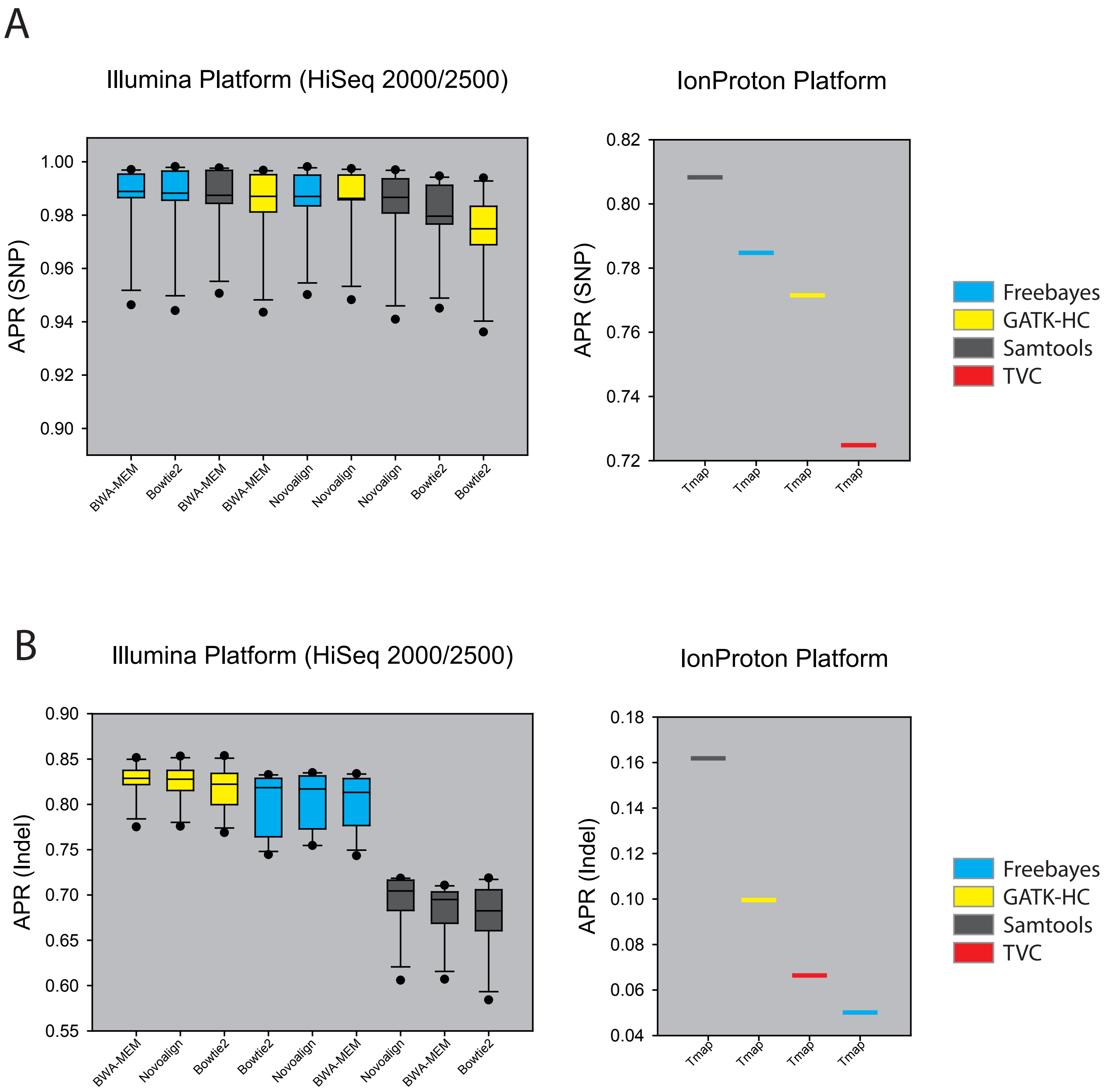

Supplementary Figure 3. Plots of exome coverage vs. APR for SNP and indel.

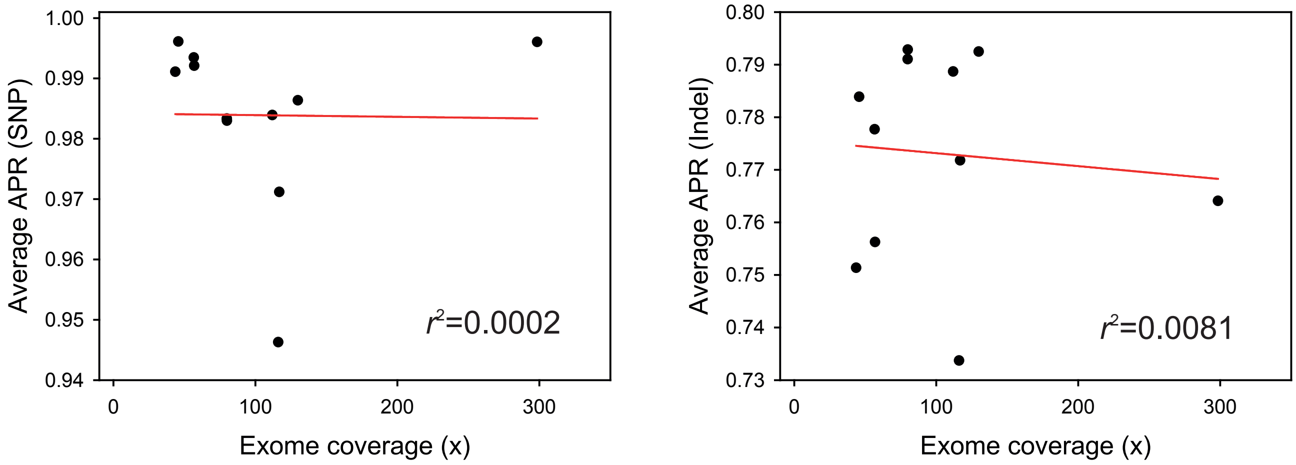

Supplementary Figure 4. Summary of variant calling performances by thirteen pipelines, based on PPV. Performances were measured by PPV rather than APR for (A) SNPs and (B) indels.

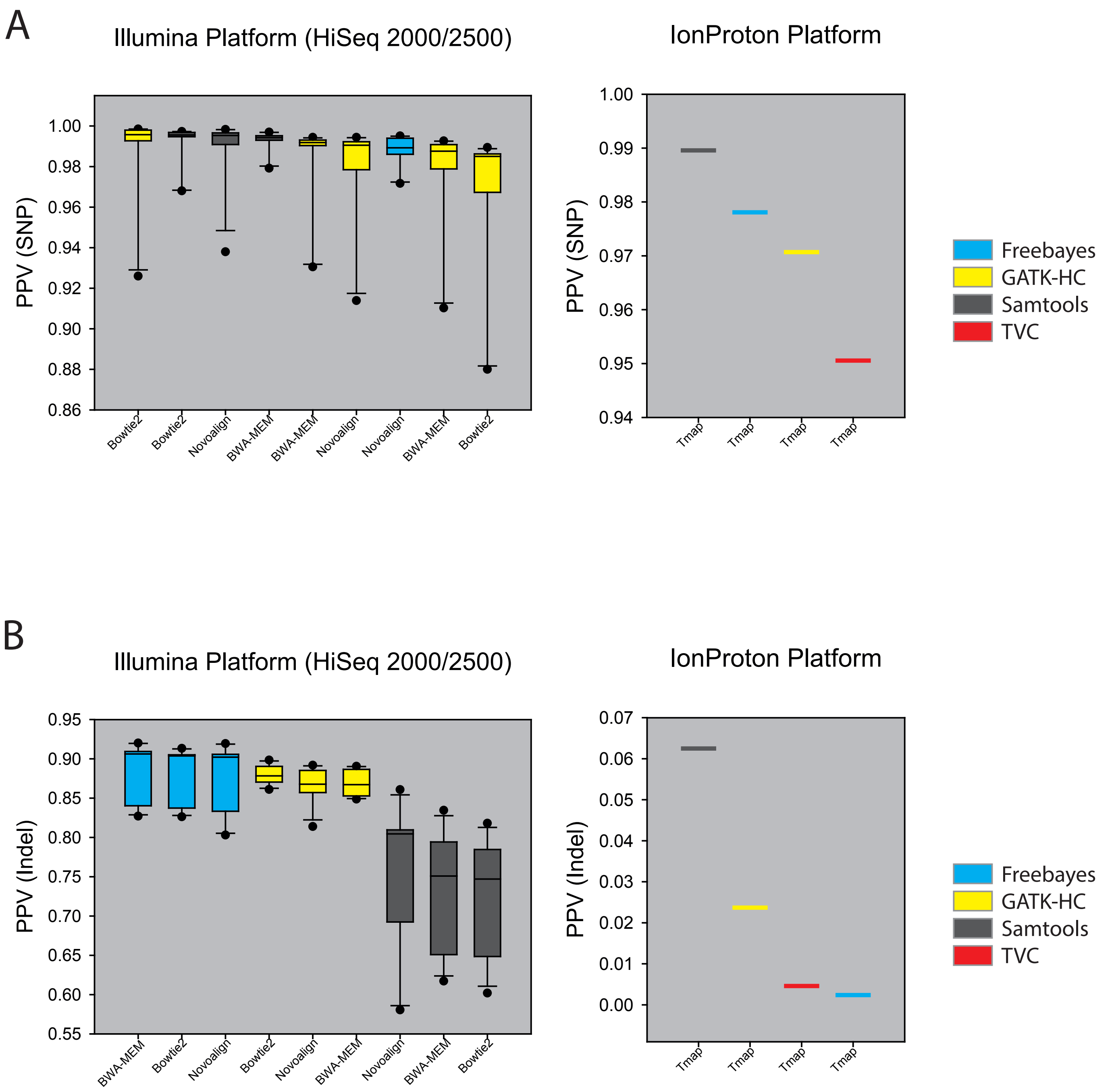

Supplementary Table 1. Summary table of performance of SNP calling from twelve data sets using thirteen pipelines.

SNP	Area under Precision-Recall curve(APR)			True Positive(TP)				False Positive(FP)		
Illumina HiSeq 2000/2500	GATK-HC	Samtools	Freebayes	# of gold standard SNP	GATK-HC	Samtools	Freebayes	GATK-HC	Samtools	Freebayes
SRR1611178-BWA-MEM	0.981	0.987	0.988	40532	39162	39524	39713	322	243	368
SRR1611179-BWA-MEM	0.981	0.987	0.988	40532	39183	39547	39739	346	229	380
SRR292250-BWA-MEM	0.944	0.954	0.947	26263	23769	23916	23884	1774	376	2354
SRR515199-BWA-MEM	0.996	0.997	0.996	32470	32304	32320	32337	181	97	257
SRR098401-BWA-MEM	0.967	0.979	0.975	25870	24640	24847	24906	1660	528	2096
SRR1611183-BWA-MEM	0.987	0.990	0.989	40532	39628	39726	39821	275	150	295
SRR1611184-BWA-MEM	0.984	0.988	0.987	40532	39392	39566	39656	375	201	521
SRX485062-BWA-MEM	0.994	0.997	0.995	17567	17468	17481	17494	170	115	231
ERR194147-BWA-MEM	0.997	0.998	0.997	17567	17500	17507	17523	135	84	379
SRX515284-BWA-MEM	0.995	0.997	0.995	17567	17482	17491	17504	141	124	220
SRX516752-BWA-MEM	0.995	0.997	0.995	17567	17471	17485	17498	122	91	208
SRR1611178-Bowtie2	0.969	0.981	0.988	40532	38151	39044	39739	227	192	556
SRR1611179-Bowtie2	0.969	0.981	0.988	40532	38193	39076	39783	242	173	606
SRR292250-Bowtie2	0.936	0.949	0.944	26263	23437	23706	23898	1873	783	3258
SRR515199-Bowtie2	0.994	0.996	0.996	32470	32168	32242	32353	135	94	469
SRR098401-Bowtie2	0.957	0.971	0.973	25870	24058	24454	24889	1502	767	3127
SRR1611183-Bowtie2	0.975	0.983	0.989	40532	38626	39206	39826	165	140	556
SRR1611184-Bowtie2	0.972	0.981	0.986	40532	38430	39044	39642	283	207	764
SRX485062-Bowtie2	0.983	0.991	0.997	17567	17004	17266	17473	60	82	241
ERR194147-Bowtie2	0.988	0.994	0.998	17567	17195	17353	17532	30	67	593
SRX515284-Bowtie2	0.983	0.992	0.997	17567	17044	17301	17487	35	55	187
SRX516752-Bowtie2	0.978	0.987	0.995	17567	16853	17135	17411	25	46	302
SRR1611178-Novoalign	0.981	0.986	0.987	40532	39134	39427	39581	307	182	240
SRR1611179-Novoalign	0.981	0.986	0.987	40532	39166	39458	39608	315	181	242
SRR292250-Novoalign	0.941	0.954	0.949	26263	23774	23861	23727	2239	145	606
SRR515199-Novoalign	0.996	0.997	0.996	32470	32313	32307	32316	181	68	158
SRR098401-Novoalign	0.966	0.978	0.975	25870	24655	24779	24717	1813	242	720
SRR1611183-Novoalign	0.987	0.989	0.989	40532	39606	39710	39743	313	136	224
SRR1611184-Novoalign	0.984	0.987	0.986	40532	39390	39536	39559	376	186	406
SRX485062-Novoalign	0.994	0.995	0.995	17567	17408	17407	17438	186	160	226
ERR194147-Novoalign	0.997	0.999	0.997	17567	17507	17524	17517	193	30	190
SRX515284-Novoalign	0.990	0.993	0.986	17567	17311	17344	17154	381	1147	210
SRX516752-Novoalign	0.989	0.991	0.992	17567	17262	17263	17339	113	139	245

SNP	Area under Precision-Recall curve(APR)				True Positive(TP)					False Positive(FP)			
Iontorrent Proton	GATK-HC	Samtools	Freebayes	TVC	# of gold standard SNP	GATK-HC	Samtools	Freebayes	TVC	GATK-HC	Samtools	Freebayes	TVC
NA12878_combine-tmap	0.772	0.858	0.819	0.777	17567	9813	12783	11861	9866	220	386	617	104

Supplementary Table 2. Summary table of performance of indel calling from twelve data sets using thirteen pipelines.

Indel	Area under Precision-Recall curve(APR)			True Positive(TP)				False Positive(FP)		
Illumina HiSeq 2000/2500	GATK-HC	Samtools	Freebayes	# of gold standard SNP	GATK-HC	Samtools	Freebayes	GATK-HC	Samtools	Freebayes
SRR1611178-BWA-MEM	0.826	0.724	0.828	3859	2833	2376	2709	369	593	270
SRR1611179-BWA-MEM	0.829	0.716	0.830	3859	2857	2376	2722	366	645	279
SRR292250-BWA-MEM	0.775	0.677	0.744	1724	1206	918	1028	151	182	92
SRR515199-BWA-MEM	0.852	0.613	0.832	2882	2501	1560	2331	320	562	293
SRR098401-BWA-MEM	0.822	0.661	0.815	1684	1376	1049	1268	169	348	110
SRR1611183-BWA-MEM	0.838	0.708	0.835	3859	2905	2312	2746	447	602	284
SRR1611184-BWA-MEM	0.832	0.709	0.829	3859	2860	2340	2711	438	606	278
SRX485062-BWA-MEM	0.831	0.688	0.797	419	365	316	311	63	170	65
ERR194147-BWA-MEM	0.843	0.713	0.791	419	373	320	315	63	115	62
SRX515284-BWA-MEM	0.819	0.696	0.777	419	369	317	307	64	170	58
SRX516752-BWA-MEM	0.827	0.672	0.774	419	365	316	300	65	196	57
SRR1611178-Bowtie2	0.821	0.721	0.828	3859	2760	2384	2707	344	628	284
SRR1611179-Bowtie2	0.822	0.712	0.828	3859	2773	2384	2711	343	669	284
SRR292250-Bowtie2	0.769	0.668	0.746	1724	1174	913	1042	141	203	103
SRR515199-Bowtie2	0.854	0.590	0.833	2882	2472	1544	2321	304	675	302
SRR098401-Bowtie2	0.818	0.646	0.820	1684	1336	1028	1287	151	348	122
SRR1611183-Bowtie2	0.834	0.695	0.833	3859	2843	2292	2725	423	629	290
SRR1611184-Bowtie2	0.827	0.696	0.830	3859	2793	2326	2709	410	647	288
SRX485062-Bowtie2	0.829	0.719	0.789	419	347	310	299	56	168	58
ERR194147-Bowtie2	0.840	0.713	0.789	419	361	311	309	50	121	60
SRX515284-Bowtie2	0.800	0.682	0.765	419	347	311	295	50	171	62
SRX516752-Bowtie2	0.794	0.664	0.762	419	330	301	283	50	199	56
SRR1611178-Novoalign	0.826	0.733	0.829	3859	2833	2381	2720	368	560	291
SRR1611179-Novoalign	0.828	0.724	0.830	3859	2850	2380	2723	370	561	283
SRR292250-Novoalign	0.776	0.692	0.756	1724	1211	928	1062	153	150	93
SRR515199-Novoalign	0.853	0.615	0.834	2882	2507	1592	2357	325	528	308
SRR098401-Novoalign	0.825	0.719	0.820	1684	1378	1064	1280	167	222	117
SRR1611183-Novoalign	0.837	0.716	0.836	3859	2902	2329	2753	450	555	289
SRR1611184-Novoalign	0.833	0.709	0.832	3859	2862	2350	2729	436	571	296
SRX485062-Novoalign	0.832	0.716	0.799	419	362	313	318	61	139	78
ERR194147-Novoalign	0.845	0.721	0.800	419	374	320	325	57	125	65
SRX515284-Novoalign	0.797	0.716	0.756	419	350	310	296	80	224	57
SRX516752-Novoalign	0.815	0.680	0.775	419	354	302	303	59	195	69

Indel	Area under Precision-Recall curve(APR)				True Positive(TP)					False Positive(FP)			
Iontorrent Proton	GATK-HC	Samtools	Freebayes	TVC	# of gold standard SNP	GATK-HC	Samtools	Freebayes	TVC	GATK-HC	Samtools	Freebayes	TVC
NA12878_combine-tmap	0.100	0.161	0.048	0.051	419	106	159	139	57	4367	66739	30122	855

Supplementary Table 3. Summary of concordance among variant callers

Illumina Platform		20 QUAL score						
		Freebayes	Freebayes∩ GATK-HC	Freebayes∩ GATK-HC∩ Samtools	Freebayes∩ Samtools	GATK-HC∩ Samtools	GATK-HC	Samtools
wes	SRR098401_bowtie2	7.11%	4.43%	82.37%	1.11%	0.62%	2.83%	1.53%
wes	SRR098401_bwa-mem	3.46%	4.19%	85.97%	0.83%	0.45%	4.07%	1.02%
wes	SRR098401_novoalign	1.41%	2.79%	87.43%	0.62%	0.44%	6.68%	0.65%
wes	SRR1611178_bowtie2	3.41%	2.01%	88.55%	1.91%	0.64%	2.21%	1.27%
wes	SRR1611178_bwa-mem	1.29%	2.13%	91.40%	1.07%	0.79%	2.22%	1.11%
wes	SRR1611178_novoalign	1.16%	1.98%	91.65%	0.98%	0.82%	2.34%	1.07%
wes	SRR1611179_bowtie2	3.39%	1.99%	88.75%	1.86%	0.55%	2.13%	1.33%
wes	SRR1611179_bwa-mem	1.33%	2.08%	91.28%	1.07%	0.79%	2.24%	1.21%
wes	SRR1611179_novoalign	1.14%	2.00%	91.69%	0.94%	0.79%	2.29%	1.15%
wes	SRR1611183_bowtie2	3.17%	2.13%	90.21%	1.21%	0.36%	1.61%	1.31%
wes	SRR1611183_bwa-mem	0.95%	2.28%	92.91%	0.51%	0.41%	1.81%	1.13%
wes	SRR1611183_novoalign	0.75%	2.14%	93.20%	0.46%	0.39%	1.99%	1.07%
wes	SRR1611184_bowtie2	3.35%	2.28%	89.73%	1.23%	0.39%	1.66%	1.36%
wes	SRR1611184_bwa-mem	1.23%	2.40%	92.29%	0.60%	0.42%	1.88%	1.19%
wes	SRR1611184_novoalign	1.01%	2.15%	92.82%	0.52%	0.37%	2.01%	1.11%
wes	SRR292250_bowtie2	7.06%	4.73%	82.25%	0.97%	0.73%	3.27%	0.99%
wes	SRR292250_bwa-mem	4.22%	4.45%	85.69%	0.62%	0.61%	3.88%	0.53%
wes	SRR292250_novoalign	1.15%	2.32%	86.86%	0.48%	0.57%	8.17%	0.44%
wes	SRR515199_bowtie2	1.80%	3.12%	91.84%	0.40%	0.27%	1.02%	1.55%
wes	SRR515199_bwa-mem	0.95%	3.15%	93.08%	0.21%	0.25%	1.16%	1.20%
wes	SRR515199_novoalign	0.62%	3.06%	93.50%	0.20%	0.19%	1.25%	1.18%
wgs	ERR194147_bowtie2	3.45%	0.26%	94.27%	0.81%	0.15%	0.45%	0.61%
wgs	ERR194147_bwa-mem	1.40%	0.48%	96.35%	0.28%	0.15%	0.85%	0.50%
wgs	ERR194147_novoalign	0.73%	0.50%	96.89%	0.12%	0.13%	1.09%	0.54%
wgs	SRX485062_bowtie2	2.40%	0.33%	93.75%	1.56%	0.24%	0.69%	1.04%
wgs	SRX485062_bwa-mem	0.69%	0.47%	96.61%	0.46%	0.12%	0.91%	0.73%
wgs	SRX485062_novoalign	0.73%	0.57%	96.70%	0.31%	0.16%	0.89%	0.64%
wgs	SRX515284_bowtie2	2.13%	0.31%	94.15%	1.60%	0.26%	0.60%	0.96%
wgs	SRX515284_bwa-mem	0.74%	0.42%	96.70%	0.41%	0.20%	0.79%	0.74%
wgs	SRX515284_novoalign	0.80%	0.36%	92.12%	0.57%	1.51%	1.81%	2.83%
wgs	SRX516752_bowtie2	2.60%	0.45%	93.02%	1.74%	0.41%	0.65%	1.12%
wgs	SRX516752_bwa-mem	0.80%	0.48%	96.60%	0.34%	0.21%	0.75%	0.82%
wgs	SRX516752_novoalign	0.93%	0.59%	95.54%	0.58%	0.31%	1.12%	0.92%

Supplementary Table 4. Comparison of three benchmarking studies that used GIAB gold standard variants.

Comparisons of this study, Cornish *et al*[1]., and Highnam *et al*[2].

	This study	Cornish *et al.*	Highnam *et al.*
**Benchmark Set**	GIAB high confident integrated pedigree calls v0.2	GIAB list	GIAB high confident calls v2.18
**Sequencing Platforms**	Illumina HiSeq 2000/2500, Ion Proton	Illumina HiSeq 2000	Illumina
**Data sets**	12 data (NA12878, Whole Exome Sequence, Whole Genome Sequence)	Single data (NA12878, Whole Exome Sequence)	single data (Simulated data or real data)
**Sequence Read Aligner**	Bowtie2-2.2.25<br>BWA-MEM-0.7.10<br>Novoalign-3.02.12<br>Tmap (for Ion Proton)	Bowtie2-2.1.0<br>BWA sampe-0.7.5a<br>BWA-MEM-0.7.5a<br>CUSHAW3-3.0.3<br>MOSAIK-2.2.3<br>Novoalign-3.02.07	Bowtie2-2.0.0-beta5<br>BWA-0.7.5a<br>BWA-MEM-0.7.5a<br>Novoalign-3.00.04
**Variant Caller**	Freebayes-0.9.18<br>GATK-HaplotypeCaller-3.3.0<br>Samtools mpileup-1.1<br>TVC-4.2.3 (for Ion Proton)	FreeBayes v9.9.2-19-g011561f<br>GATK-HaplotypeCaller-2.7-2<br>GATK-UnifiedGenotyper-2.7-2<br>Samtools mpileup-0.1.19<br>SNPSVM-0.01	GATK HaplotypeCaller-3.1-1<br>GATK UnifiedGenotyper-3.1-1<br>Samtools mpileup v0.1.18<br>Isaac v01.13.06.20
**Benchmark Method**	Area under Precision-Recall curve(APR)	Positive Predictive Value (PPV), Sensitivity separately	Positive Predictive Value (PPV)
**Reported Best Pipeline**	**SNP** : BWA-MEM + Samtools mplieup<br>**Indel** : BWA-MEM + GATK-HaplotypeCaller (SNP and Indel were analyzed separately)	Novoalign3 + GATK-UnifiedGenotyper (SNP was analyzed only)	Novoalign3 and GATK HaplotypeCaller (SNP and Indel were combined for this analysis)

Table references

1 Cornish, A. & Guda, C. A comparison of variant calling pipelines using Genome in a Bottle as a reference. *BioMed Research International* (in press).

2 Highnam, G. *et al.* An analytical framework for optimizing variant discovery from personal genomes. *Nature Communications* **6** (2015).
